# Genome-Scale Mapping Reveals Complex Regulatory Activities of RpoN in Yersinia pseudotuberculosis

**DOI:** 10.1128/mSystems.01006-20

**Published:** 2020-11-10

**Authors:** A. K. M. Firoj Mahmud, Kristina Nilsson, Anna Fahlgren, Roberto Navais, Rajdeep Choudhury, Kemal Avican, Maria Fällman

**Affiliations:** a Department of Molecular Biology, Laboratory for Molecular Infection Medicine Sweden (MIMS), Umeå Centre for Microbial Research (UCMR), Umeå University, Umeå, Sweden; Pacific Northwest National Laboratory

**Keywords:** ChIP-Seq, RNA-seq, RpoN, *Yersinia*, antisense binding, genome mapping, sigma factor regulation, sigma factors, transcription factors, transcriptional regulation

## Abstract

RpoN, an alternative sigma factor commonly known as σ^54^, is implicated in persistent stages of Yersinia pseudotuberculosis infections in which genes associated with this regulator are upregulated. We here combined phenotypic and genomic assays to provide insight into its role and function in this pathogen. RpoN was found essential for Y. pseudotuberculosis virulence in mice, and *in vitro* functional assays showed that it controls biofilm formation and motility. Mapping genome-wide associations of Y. pseudotuberculosis RpoN using chromatin immunoprecipitation coupled with next-generation sequencing identified an RpoN binding motif located at 103 inter- and intragenic sites on both sense and antisense strands. Deletion of *rpoN* had a large impact on gene expression, including downregulation of genes encoding proteins involved in flagellar assembly, chemotaxis, and quorum sensing. There were also clear indications of cross talk with other sigma factors, together with indirect effects due to altered expression of other regulators. Matching differential gene expression with locations of the binding sites implicated around 130 genes or operons potentially activated or repressed by RpoN. Mutagenesis of selected intergenic binding sites confirmed both positive and negative regulatory effects of RpoN binding. Corresponding mutations of intragenic sense sites had less impact on associated gene expression. Surprisingly, mutating intragenic sites on the antisense strand commonly reduced expression of genes carried by the corresponding sense strand.

**IMPORTANCE** The alternative sigma factor RpoN (σ^54^), which is widely distributed in eubacteria, has been implicated in controlling gene expression of importance for numerous functions including virulence. Proper responses to host environments are crucial for bacteria to establish infection, and regulatory mechanisms involved are therefore of high interest for development of future therapeutics. Little is known about the function of RpoN in the intestinal pathogen Y. pseudotuberculosis, and we therefore investigated its regulatory role in this pathogen. This regulator was indeed found to be critical for establishment of infection in mice, likely involving its requirement for motility and biofilm formation. The RpoN regulon involved both activating and suppressive effects on gene expression which could be confirmed with mutagenesis of identified binding sites. This is the first study of its kind of RpoN in Y. pseudotuberculosis, revealing complex regulation of gene expression involving both productive and silent effects of its binding to DNA, providing important information about RpoN regulation in enterobacteria.

## Abstract (precis)

The alternative sigma factor RpoN (σ^54^), which is widely distributed in eubacteria, has been implicated in controlling gene expression of importance for numerous functions including virulence. Proper responses to host environments are crucial for bacteria to establish infection, and regulatory mechanisms involved are therefore of high interest for development of future therapeutics. Little is known about the function of RpoN in the intestinal pathogen Y. pseudotuberculosis, and we therefore investigated its regulatory role in this pathogen. This regulator was indeed found to be critical for establishment of infection in mice, likely involving its requirement for motility and biofilm formation. The RpoN regulon involved both activating and suppressive effects on gene expression which could be confirmed with mutagenesis of identified binding sites. This is the first study of its kind of RpoN in Y. pseudotuberculosis, revealing complex regulation of gene expression involving both productive and silent effects of its binding to DNA, providing important information about RpoN regulation in enterobacteria.

## INTRODUCTION

During infection of a host, bacteria are exposed to rapid changes in the environment, such as changes in temperature, pH, osmolarity, and nutrient levels, and immune cell attacks. Bacteria usually cope with these types of changes through stress responses, alterations of their gene expression that are adaptive to the new environment (1). This type of infection-associated transcriptomic reprogramming was obvious in a previous *in vivo* transcriptomic study of Yersinia pseudotuberculosis isolated from cecal lymphoid compartments of infected mice (2). In that model, plasmid-carried virulence genes known to be necessary for tissue invasion and resistance toward initial attacks from phagocytes were highly expressed during the early phase of the infection. After about one-and-a-half months of symptomless infection, the expression pattern had changed so that genes encoding proteins involved in adaption and resistance to different types of stresses dominated, while expression of the plasmid-carried virulence genes was considerably reduced. This highlights the importance for bacteria of both adapting to new environments and the regulatory mechanisms involved. Hence, increasing our understanding of bacterial function during infection is of great interest. Mechanisms of bacterial adaptation inform choices of potential targets for new antibiotics, with gene products required to maintain infections considered more promising than those of classical virulence genes.

Transcriptional reprogramming is commonly controlled by various transcriptional regulators that are activated in response to external signals. A major class of transcriptional regulators are sigma factors, which upon activation associate with the core RNA polymerase (RNAP), promoting its binding to specific initiation sites and subsequent open complex formation for transcription of downstream genes (3). There are different types of sigma factors in bacteria, where RpoD or sigma 70 (σ^70^) is the primary and housekeeping sigma factor active during exponential growth (4–6). Other alternative sigma factors such as RpoE, RpoS, RpoH, and RpoN, which recognize promoter sequences distinct from that of RpoD, regulate transcription under specific conditions, allowing expression of genes required for the bacteria to cope with and adapt to particular situations (5). One of the sigma factors that attracted interest during the analysis of data from our previous *in vivo* transcriptomic analysis of Y. pseudotuberculosis was RpoN or sigma 54 (σ^54^), which together with many of its associated proteins, including activators and modulating proteins, was upregulated during the persistent stages of infection, when the expression of genes important for adaptation to the tissue environment dominated (2). RpoN has been reported to control regulation of genes involved in nitrogen metabolism, flagella, and motility, but biofilm formation and quorum sensing can also be affected in *rpoN* mutant strains (7–10). In some species, RpoN also influences regulation of type III and type VI secretion (8, 11–13), and there are many reports implicating RpoN as a regulator of bacterial virulence (9, 14, 15).

RpoN is structurally and functionally distinct from other sigma factors in that transcription initiation commonly depends on its binding to activating proteins termed bacterial enhancer-binding proteins (EBPs) (6). These EBPs use ATP catalysis to remodel RNAP DNA binding to initiate transcription (16). The affinity of RpoN for the core RNAP is higher than that of most other RpoD-related alternative sigma factors, allowing it to compete efficiently for RNAP binding. Regulation by RpoN can be either direct or indirect via activation of different positive or negative regulators, including other sigma factors (5, 17, 18). Compared with other sigma factors, direct cross talk whereby different sigma factors regulate the same gene is particularly high for RpoN and is also commonly seen for genes that encode proteins involved in complex processes with different levels of regulation, such as adaption, chemotaxis, adhesion, and protein secretion (19). Adding to the complexity is the variation between different bacteria in the specific signals regulating RpoN and the specific downstream outcomes. One example is biofilm formation, where a deletion of the *rpoN* gene results in severe effects on the capacity to form biofilms in many bacteria but where the opposite is seen in some other bacteria (20, 21).

This study investigated the regulatory role of RpoN in Y. pseudotuberculosis, which had not previously been addressed in detail. Neither has the RpoN regulon been defined for this pathogen. The results revealed that RpoN is crucial in Y. pseudotuberculosis to establish infection and is required for biofilm formation and motility. Chromatin immunoprecipitation coupled with next-generation sequencing (ChIP-seq) was used to determine genome-wide binding and revealed more than 100 RpoN binding sites with both inter- and intragenic locations. Transcriptomic data from bacteria lacking *rpoN* implied a complex regulatory network with direct or indirect effects. Matching the locations of ChIP peaks with transcriptomic data allowed retrieval of more than 130 genes potentially regulated by RpoN, some novel and some known from previous studies. Mutagenesis of selected RpoN binding sites confirmed both activating and suppressive roles of upstream intergenic RpoN binding. This was not seen for sites of intragenic binding to the sense strand. In contrast, mutation of RpoN binding motifs on the antisense strand commonly resulted in suppressed expression of the gene on the sense strand, implicating a novel regulatory mechanism.

## RESULTS AND DISCUSSION

### RpoN participates in regulation of Y. pseudotuberculosis biofilm formation and motility and is required for virulence.

**FIG 1:**
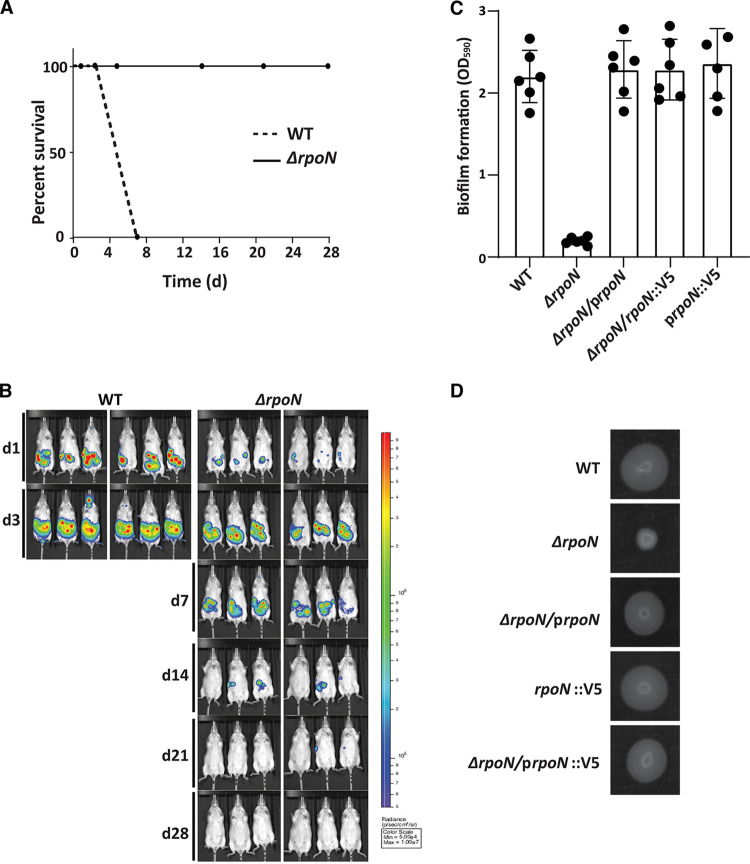
RpoN is required for Y. pseudotuberculosis virulence and essential for motility and biofilm formation. (A) Female FVB/N mice (*n* = 6) were orally infected with 4 × 10^8^ CFU/ml of Y. pseudotuberculosis WT (dashed line) or the isogenic Δ*rpoN* mutant strain (solid line). Data are presented as percent live mice during the course of infection (0 to 28 days). (B) IVIS *in vivo* images showing anesthetized mice during indicated days of infection. Pseudocolors show levels of light emitted by bioluminescent bacteria. Mice infected with Y. pseudotuberculosis WT became sick at day 5 to 7 and were sacrificed. (C) Biofilm formation by Y. pseudotuberculosis WT and corresponding strains lacking the *rpoN* gene (Δ*rpoN*), Δ*rpoN* complemented in *trans* with a plasmid expressing RpoN (Δ*rpoN*/p*rpoN*), V5-tagged *rpoN* in the chromosome under its native promoter (*rpoN*::V5), and Δ*rpoN* complemented in *trans* with a plasmid expressing V5-tagged rpoN (Δ*rpoN*/p*rpoN*::V5). Biofilm mass was determined by dissolving biofilm material and measuring the OD_590_. Data are presented as means ± SEM for six individual experiments. (D) Motility in agarose by the same strains as indicated in panel C. One representative experiment of three replicates is shown.

To reveal the importance of RpoN in Y. pseudotuberculosis virulence, an *rpoN* deletion mutant strain was constructed to be used in mouse infection studies. The resulting Δ*rpoN* strain was tested for eventual defects in growth at 26°C reaching stationary phase and during virulence induction at 37°C and also tested for acid sensitivity (see Fig. S1A and B in the supplemental material). The mutant strain was here found to grow and tolerate acid to the same extent as the wild-type (WT) strain, excluding possible negative effects on the ability of the mutant to reach and proliferate at the infection site caused by the passage through the acidic environment in the stomach. The halted growth that occurs when shifting to 37°C and depleting extracellular Ca^2+^ is a known consequence of increased expression of the virulence plasmid (22), which was seen also for the Δ*rpoN* strain. In accordance with prior results using this acute virulence model of Y. pseudotuberculosis oral infection, the WT strain initially colonized all infected mice, and all showed clear signs of disease and succumbed at day 5 to 7 postinfection (Fig. 1A). The Δ*rpoN* strain, on the other hand, had colonized all mice to a limited extent 1 day after oral infection and showed increased colonization at day 3, but then the infection declined, and the majority were cleared at day 14 to 21. At 28 days postinfection (dpi), all mice had cleared the infection (Fig. 1B and Fig. S1C). None of the mice infected with the Δ*rpoN* strain showed signs of disease during the infection period, indicating clear attenuation. We also employed the Y. pseudotuberculosis infection model for persistent infection of the cecal lymphoid compartment, where a fraction of mice infected with low doses of the WT strain develop acute disease and another fraction carry bacteria in cecal tissue for prolonged times without showing symptoms of disease (23). Upon low-dose oral infection with the Δ*rpoN* strain, only 75% of infected mice were initially colonized compared with mice infected with the corresponding WT strain, all of which were colonized (Fig. S1C). Further, none of the Δ*rpoN* strain-infected mice developed acute infection, whereas the fraction causing symptomless persistent infection was almost similar to that of the WT strain (Fig. S1C), suggesting a role for this regulator during the early stages of infection. We next tested the Δ*rpoN* strain in different phenotypic assays and found that the mutant strain was deficient in biofilm formation and motility (Fig. 1C and D). The biofilm and motility phenotypes could be complemented by expressing RpoN in *trans* (Δ*rpoN*/p*rpoN*). Hence, it is obvious that the Δ*rpoN* mutant strain has limited functional capacity that likely contributes to the observed attenuation in virulence. Together, these data indicate a pivotal role for RpoN in Y. pseudotuberculosis virulence.

10.1128/mSystems.01006-20.1 FIG S1 High- and low-dose infection by Y. pseudotuberculosis WT and Δ*rpoN* in female FVB/N mice. (A) Growth curves for WT and Δ*rpoN* strains in LB at 26°C and during virulence induction at 37°C. (B) Acid sensitivity test. Overnight cultures were diluted to 10^8^ CFU/ml and exposed to different pHs for 2 h. Tenfold serial dilutions were spotted on LA plates. (C) Mice (one experiment, *n* = 6) were orally infected with a mean of 4 × 10^8^ CFU/ml for high-dose infection, and for low-dose infection, mice (one experiment: WT, *n* = 40, and Δ*rpoN*, *n* = 20) were infected with a mean of 0.8 × 10^6^ CFU/ml. Mice that succumbed to acute infection were immediately sacrificed. The percentage of mice developing acute infection, the mice with symptom-free infection, and the fraction that cleared the infection were determined at 28 days after infection and related to total number of mice initially infected. Download FIG S1, PDF file, 1.4 MB. Copyright © 2020 Mahmud et al. 2020 Mahmud et al. This content is distributed under the terms of the Creative Commons Attribution 4.0 International license.

### RpoN binds to multiple sites on the Y. pseudotuberculosis chromosome.

Since previous studies of RpoN-mediated events have indicated variation in the regulatory network between different bacterial species, and since studies of RpoN in Y. pseudotuberculosis are limited, we set out to uncover the regulatory mechanisms involved. As a first step, we employed ChIP-seq to determine the RpoN DNA binding sites in Y. pseudotuberculosis. ChIP-seq strongly depends on an appropriate antibody, and we chose to fuse the C terminus of RpoN to the 3×V5 epitope (14 amino acids found in C-terminal sequence of the P and V proteins of simian virus 5), which has been shown to function in similar approaches in other bacteria (24). We generated a vector construct to overexpress RpoN-V5 (p*rpoN*::V5) and also a strain expressing RpoN-V5 from its native promoter, by inserting the sequence for the V5 tag in the 3′ end of the *rpoN* gene (*rpoN*::V5). Repeating the assays for biofilm formation and motility and including the strains expressing V5-tagged RpoN (Δ*rpoN/*p*rpoN*::V5 and the *rpoN*::V5 strains) showed that the V5 tag did not interfere with RpoN function (Fig. 1C and D). It is commonly assumed that RpoN levels and binding to RNAP and to DNA are relatively stable and that the EBPs play the regulatory role. However, our finding of differential expression of *rpoN in vivo* compared with its expression level *in vitro* (2), and the possibility of chromosomal structural changes influencing gene expression under certain conditions (2, 25, 26), prompted us to map the binding of RpoN in bacteria subjected to more than one condition. The conditions used were exponential growth at 26°C, where RpoN-V5 either is overexpressed in *trans* (p*rpoN*::V5) or is expressed in *cis* under its native promoter (*rpoN*::V5), the latter to ensure proper stoichiometry and thereby avoid side effects of competition with other sigma factors. We also included samples subjected to virulence-inducing conditions, with a shift to 37°C and depletion of extracellular Ca^2+^ for 75 min using the *rpoN*::V5 strain.

**FIG 2:**
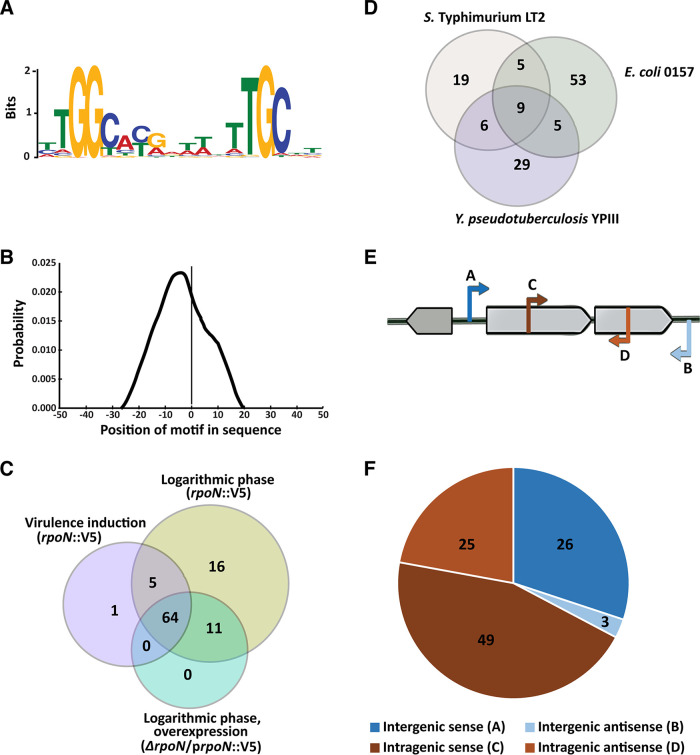
ChIP-seq identifies RpoN binding sites in Y. pseudotuberculosis genome. (A) RpoN consensus binding motif in Y. pseudotuberculosis derived from 119 ChIP-seq peaks, determined using BCRANK (E value = 1.8e^−110^). (B) Position of motifs relative to peak centers calculated using the Centrimo tool, MEME suite. The graph indicates the average density of motif position for all 119 motif-containing regions, using 10-bp bins from position −50 to +50 relative to the ChIP-seq peak. For identification of an RpoN consensus binding motif (A) and its position (B), all 3 ChIP-seq data sets (*rpoN*::V5 during logarithmic growth and virulence induction and p*rpoN*::V5 during logarithmic growth) were used with a stringent cutoff of false-discovery rate (FDR) (1E^−30^). (C) Venn diagram showing the number of RpoN binding sites identified by ChIP sequencing of Y. pseudotuberculosis under different growth conditions. (D) Venn diagram showing number of RpoN binding sites with homologous genomic positions in Y. pseudotuberculosis, E. coli, and *S*. Typhimurium. Those genes which were found to be conserved based on function and phylogenetic distance ratios among all three bacteria and whose corresponding gene was reported (by a ChIP-seq study) to be regulated by RpoN were included in the Venn diagram. (E) Schematic illustration of four classes of RpoN binding sites based on their positions in relation to gene coding sequences. (F) Distribution of each class of RpoN binding site.

The resulting ChIP-seq data were subjected to a high-stringency bioinformatic analysis with a cutoff of a 2.5-fold difference over genomic noise. The analysis that was done for all samples individually identified totally 119 ChIP-seq peaks representing putative sites for RpoN binding. The number of peaks was in the range previously shown for Escherichia coli, Salmonella enterica serovar Typhimurium, and Vibrio cholerae (8, 27, 28). Some of the peaks were narrow and distinct, covering 200 to 300 nucleotides, whereas others were relatively broad and covered 300 to 800 nucleotides (see Table S1 in the supplemental material). All 119 predicted peaks were used to identify a common sequence motif in the ±50-bp regions of the peak center. We identified a motif that resembled the RpoN −24/12 promoter element found in other bacteria (27, 29, 30) (Fig. 2A). To determine motif strength, defined by level of enrichment of RpoN within a binding site, NN-GG-N9-TGC-NN was used as the base for position-weight matrix calculations. The identified motifs had PSSM (position-specific scoring matrix) scores ranging from 3 to 12, and the motif sequences were found to cover the peak center area (Fig. 2B). The fact that the majority of the predicted motifs are found around the peak center implies that they are genuine RpoN binding sites.

10.1128/mSystems.01006-20.4 TABLE S1 ChIP-seq peaks and predicted motif coordinates. Download Table S1, PDF file, 0.8 MB. Copyright © 2020 Mahmud et al. 2020 Mahmud et al. This content is distributed under the terms of the Creative Commons Attribution 4.0 International license.

We set a cutoff for the PSSM of scores of >7 for high-confidence peaks, which yielded 103 peaks encompassing 112 binding sites (Fig. 2C; Tables 1 and 2). There were relatively few ChIP-seq peaks without motifs (16 out of 119; Table S2) compared with the findings in some other studies (24, 27, 28). This probably reflects the high-stringency analysis used, which limits the number of false positives commonly found in ChIP data from highly transcribed regions (31, 32). The majority of the peak-associated RpoN binding sites were found in bacteria expressing RpoN-V5 from its native promoter during logarithmic growth. The corresponding samples from bacteria overexpressing RpoN-V5 lacked six of those peaks, and even more peaks were missing from the samples from virulence-inducing conditions. The reasons for these differences are not obvious, but in the case of peaks missing in bacteria induced for expression of the virulence plasmid, the availability of exposed sites might have been affected by structural changes in the chromosome *per se* that can be part of mechanisms suppressing chromosomal gene expression (2, 25, 26). In bacteria overexpressing RpoN-V5, there might be a saturation effect at high RpoN-V5 concentrations, with the precipitation of RpoN molecules not associated with any binding site that dilutes samples resulting in relatively less precipitated DNA where DNA fragments from low-abundance binding sites are missed. Notably, unlike other sigma factors, RpoN can bind its DNA sequence without RNAP, although the binding is 10-fold less efficient than the RpoN:RNAP complex (33). ChIP-seq should therefore also detect RpoN-DNA interactions that are independent of active transcription.

**TABLE 1:** Intergenic sense (A) and antisense (B) RpoN binding sites^*a*^

Peak	PC	PSL	PSI	PSO	Motif sequence	Strand (+/−)	PSSM	Associated σ factor(s)	Downstream gene(s)	Distance from PC
Intergenic sense (A sites)										
IGS1	117503	10.6	13.9	9.3	CTGGCTTGATTTATGCAA	+	10.26	NA	YPK_0100	NA
IGS2	353423	9.6	11.0	8.7	TTGGCTTGTTTATTGCTT	−	11.14	NA	YPK_0329	NA
IGS3	779812	10.5	11.5	8.6	CTGGCACGTTAGTTGCAT	+	11.29	NA	YPK_0719	NA
IGS4	1127431	7.7	5.8	7.1	TAGGCTTGGATCTTGCTT	+	10.15	NA	YPK_1001	NA
IGS5	1384233	13.1	14.2	9.3	TTGGCACGCTAACTGCAT	+	10.82	NA	YPK_1257, YPK_1258^S^	NA
IGS6	1469648	5.2	NA	3.4	CTGGCATGAGTCGTGCTC	−	10.08	σ^24,54,70^	YPK_1333	29, −70
IGS7	1700633	7.4	4.9	9.7	CTGGCACGATTACTGCAA	+	10.75	σ^54,70^	YPK_1538	69
IGS8	1770585	5.9	4.4	7.3	CTGGCACGATCTTTTCAT	−	10.32	σ^70,54^	YPK_1600^E,S^	−23
IGS9	2092867	16.8	8.4	6.5	TGGGCACGCATTTTGCCT	−	10.54	σ^70,28,54^	YPK_1886	−28, −127
IGS10	2101765	16.5	15.0	13.5	TTGGCACGACTCTTGATT	+	11.44	σ^70,54^	YPK_1894^E,S,V^	−37
IGS11	2153679	4.7	5.4	NA	CTGGTGCAGATTTTGCAG	−	9.6	σ^70,24,54^	YPK_1940	−4, −14
IGS12	2194598	16.6	16.8	13.8	TTGGCACGATAACTGCTT	−	10.96	σ^38,70,54^	YPK_1974	−14, −43
IGS12	2194598	16.6	16.8	13.8	AGGGGTTAATATTTGCGT	+	9.34	σ^54,70,38^	YPK_1975	61, 141
IGS13	2298808	7.2	3.5	5.2	TTGGGATGGTTGATGCAT	−	10.22	σ^54,70^	YPK_2070	39
IGS13	2298808	7.2	3.5	5.2	TTGGAATAGTTGATGCAT	−	9.94	σ^54,70^	YPK_2070	39
IGS14	2473234	4.3	NA	4.5	GTGGCACGAAAGCTGCTG	−	10	σ^54,24,38,70^	YPK_2229^E,S^	12, 38, 52
IGS15	2990747	16.9	7.7	8.0	ATGGCATCTTATTTGCTC	+	10.51	σ^54,38,70^	YPK_2707	6, 31
IGS16	3172389	16.6	14.6	11.7	TTGGCATGGCAATTGCGC	−	10.63	NA	YPK_2873	NA
IGS17	3205379	15.4	15.3	11.8	ATGGCATGATAATTGCTT	−	11.42	σ^24,54,70^	YPK_2908^E,S^	44, −44
IGS17	3205379	15.4	15.3	11.8	TTGGCATAGGAATTGCCT	+	10.63	σ^54,24,70^	YPK_2909^E,S^	66, 104
IGS18	3225401	16.1	9.7	8.8	TTGGTCCAAGAGTTGCTT	+	10.11	NA	YPK_2927	NA
IGS19	3300593	8.9	7.7	6.2	TTGGCGTGTTTTTTGCAT	+	11.21	σ^54,70^	YPK_3001	39
IGS20	3311541	12.6	13.3	10.6	CTGGCACAAACCTTGCAT	+	10.73	σ^54,70,38,24^	YPK_3010^S^	15, 17, 23
IGS20	3311541	12.6	13.3	10.6	ATGGCTGCTTTACTGCCT	+	9.24	σ^54,70,38,24^	YPK_3010^S^	15, 17, 23
IGS20	3311541	12.6	13.3	10.6	CAGGGCTATATTCCGCTT	+	8.12	σ^54,70,38,24^	YPK_3010^S^	15, 17, 23
IGS21	3520103	13.5	9.2	10.8	ATGGCATAGCCTTTGCTT	−	10.36	NA	YPK_3220^E,S^	NA
IGS22	3650370	3.6	NA	2.7	TTGGCATGGTACTTGCAA	−	11.11	σ^24,70,54^	YPK_3329	−29, −39
IGS23	3651607	11.2	6.3	5.4	TTGTCAGGTTTCGTGCTG	−	10.18	σ^32,24,54,70^	YPK_3330	−40, −9
IGS23	3651607	11.2	6.3	5.4	CTGGAACAGCTCTTGCTT	+	10.08	σ^70^	YPK_3331	60
IGS24	4250134	12.0	13.5	10.1	TTGGCACGTTTCTTGTAA	−	10.43	NA	YPK_3857^S,E^	NA
IGS25	4358596	5.6	3.3	4.0	TTGGCGCGATTCATGCCT	−	10.45	σ^70,38^	YPK_3950	−154, 136
IGS25	4358596	5.6	3.3	4.0	GAGGCATGAATCGCGCCA	+	8.2	NA	YPK_3951	NA
IGS26	4621308	10.7	13.2	9.0	TTGGTGCCTCATTTGCGC	−	9.18	NA	YPK_4188	NA
IGS26	4621308	10.7	13.2	9.0	TTGGCATAGATTTCGCAA	+	9.96	σ^70,28,24^	YPK_4189^E,S,V^	−112, −169, 178
Intergenic antisense (B sites)										
IGAs1	2892926	5.4	5.2	4.6	TTGGCGTGAATTTTGCGC	−	10.68	σ^32,28^	YPK_2636	−17, 14
IGAs2	3172389	16.6	14.6	11.7	AGGGCATAAACGGTGCAA	+	8.71	NA	YPK_2874	NA
IGAs3	4432739	9.6	11.8	8.2	CTGGCACGCTAAGTGCAA	−	10.25	NA	YPK_4019	NA

a Abbreviations: IGS, intergenic sense; IGAs, intergenic antisense; PC, peak center base pair; PSL, peak strength logarithmic phase; PSI, peak strength induction; PSO, peak strength over expression of RpoN; PSSM, position-specific score matrix; X^E/S/V^, prefix indicating existence of RpoN binding motif in similar location in relation to orthologous genes in E. coli (E), V. cholerae (V), or *S*. Typhimurium (S); NA, not applicable.

**TABLE 2:** Intragenic sense (C) and antisense (D) RpoN binding sites^*a*^

Peak	PC	PSL	PSI	PSO	Motif sequence	Strand (+/−)	PSSM	Associated σ factor(s)	CDS	Downstream gene(s)	Distance from PC
Intragenic sense (C sites)											
IrGS1	173082	7.9	3.8	4.3	ATGGCACGATTAATGCCA	+	10.3	σ^54,32,28^	YPK_0145^S^	YPK_0147, YPK_0148, YPK_0149	52, 140
IrGS2	506362	6.0	5.9	4.9	ATGGTCCGTTTATTGCGT	+	10.2	σ^54,32,24^	YPK_0464	YPK_0465	85, 123
IrGS3	535257	3.8	NA	NA	ATGGCACAAAATGTGCTG	−	10.1	σ^54,24,32^	YPK_0482	YPK_0481	57, 187
IrGS4	613541	10.8	5.5	7.0	TTGGCTCGATTTATGCGT	−	10.7	σ^54,70,24^	YPK_0560	YPK_0559	4, 21
IrGS5	653552	6.5	5.3	3.3	ATGGCGCAGTTTATGCGT	+	9.99	σ^54,32^	YPK_0589	YPK_0591, YPK_0592	42
IrGS6	818023	4.2	NA	NA	GCGGCATGGTTGTTGCAA	−	9.99	σ^38,54,70,28,32^	YPK_0744	NA	−16, 35, 36, 39
IrGS7	821601	4.7	2.9	3.3	GCGGCATGGTTGTTGCAA	−	9.99	σ^54,28,32^	YPK_0746	YPK_0745	175, 132
IrGS8	940388	4.1	NA	3.4	TTGGCATTGTTGATGCTC	+	10.1	σ^24,28,54,38^	YPK_0824	YPK_0825	−26, −26, 64
IrGS9	1141660	6.5	4.2	3.4	TTGGTATGATTTATGCCT	+	10.6	σ^54,70,38,32,24^	YPK_1016	YPK_1018	35, 36, 76, 78
IrGS10	1225391	3.3	NA	NA	TTGGCTGAAAACTTGCAG	+	9.88	σ^54,24^	YPK_1084	YPK_1085	28
IrGS11	1308478	3.5	NA	NA	CAGGCATGGATTATGCAA	+	9.59	σ^70,24,32,38,54^	YPK_1170	YPK_1171, YPK_1172	−4, −9, −19, −27
IrGS12	1475860	6.0	4.2	3.5	TTGGCCCACGCCTTGCTT	+	9.92	σ^24,54,28^	YPK_1338	YPK_1339	−13, 16
IrGS13	1528613	6.6	4.6	3.7	CCGGCACGTTTTGTGCAG	+	9.86	σ^24,54,38^	YPK_1386	YPK_1387	−19, 14
IrGS14	1605153	18.0	9.3	9.0	TTGGTCTGACTTTTGCTT	−	10.4	σ^54,32,38^	YPK_1454	YPK_1452, YPK_1453	21, 45
IrGS15	1930700	3.6	NA	2.8	TTGGCGGGAATATTGCTT	−	10.7	σ^54,28^	YPK_1742	NA	86
IrGS16	2058280	6.7	4.8	3.7	TTGGTATGTTATTTGCAA	+	10.7	σ^24,28,54,38,70^	YPK_1853	NA	−44, −44, 22, 135
IrGS16	2058280	6.7	4.8	3.7	TAAGCATGAAACGTGCAT	+	9.04	σ^32,70,54,38,28^	YPK_1853	NA	−44, −44, 22, 135
IrGS17	2211354	7.4	3.4	4.5	TTGGTACATTTATTGCGC	−	10.4	σ^24,54,38^	YPK_1988	NA	−49, 123
IrGS18	2408285	4.5	NA	3.6	CTGGCACGTCTGATGCAA	+	9.98	σ^32,54^	YPK_2169	YPK_2170, YPK_2171	−19
IrGS19	2536664	7.6	4.1	5.5	TTGGCATGGTAATTGAAT	−	10.7	σ^24,54,28,32^	YPK_2282	YPK_2281	−44, 7, 8
IrGS20	2598060	7.1	4.7	6.2	TTGGCACAAAGCTTGCTC	−	10.6	σ^32,54,28,24^	YPK_2364	YPK_2362	−16, 4, 15
IrGS21	2599974	6.9	7.5	6.3	CTGGCACGTAAATTGTAT	−	9.67	σ^24,70,54,28,32^	YPK_2365	YPK_2364	−30, −50, 100, 112
IrGS22	2663968	11.3	6.5	5.8	TTGGCCCGCTTCTTGCGC	−	10.6	σ^70,24,54,28,32^	YPK_2431^V^	YPK_2429^S^, YPK_2430	−29, −31, 32, 46
IrGS23	2760688	8.9	6.0	3.7	ACGGAGCACTTCTTGCAT	−	9.25	σ^54,24,32^	YPK_2519	YPK_2517, YPK_2518	69, 120
IrGS24	2815385	3.0	NA	NA	ACGGTATAATTATTGCGT	−	10	σ^28,54,70^	YPK_2564	NA	−1, 70
IrGS25	2870433	3.2	NA	NA	ATGGTATAAAAATTGCGC	−	9.85	NA	YPK_2615	NA	NA
IrGS26	2978162	15.7	8.2	7.7	TTGGCACGATTGATGCTC	−	10.9	σ^70,28,38,54^	YPK_2696	YPK_2695	−26, −53, −86
IrGS27	3075166	4.1	3.3	2.2	CTGGCTTAATGTTTGCAT	+	10.5	NA	YPK_2785	YPK_2786, YPK_2787	NA
IrGS28	3195552	4.4	NA	NA	CTGGCAAAAATTATGCCC	+	9.63	σ^32,38,28,54,24^	YPK_2901	YPK_2902	−7, −24, −44, 146
IrGS29	3266581	3.4	NA	NA	CTGGAACGTTATTTGCAG	−	10.4	σ^38,32,54,24,70^	YPK_2968	YPK_2967	−1, −13, 20, 148
IrGS30	3276693	3.3	NA	3.1	TTGGCATCAAAGTTGCCG	+	10.3	σ^54,38,24^	YPK_2976	YPK_2977	67, 147
IrGS31	3386998	4.7	NA	2.9	TCGGCCCGTTTATTGCTC	−	10.4	σ^54,28,32,38,28^	YPK_3080	YPK_3079	44, 86, 91
IrGS32	3448213	2.6	2.4	NA	GTGGCGCGTTATTTGCGT	−	10.5	σ^28,24^	YPK_3152	NA	80, 152
IrGS33	3491164	5.6	4.1	3.5	GTGGAACAGGTTTTGCAC	−	9.94	σ^28,32,54,70^	YPK_3192	YPK_3191	−5, −10, 181
IrGS34	3558532	6.5	5.8	4.2	TTGGTACGTTACTTGCTC	+	10.7	σ^28,54,32,38,24^	YPK_3253	NA	−44, 73, 89, 95
IrGS35	3621908	4.7	5.0	4.4	CTGGCAAATTTTCTGAAA	−	9.43	σ^54,32,28^	YPK_3308	YPK_3307	2, 57
IrGS36	3658670	6.0	5.5	3.9	TTGGCACAAAACGTGCGT	+	10.4	σ^54,32,38^	YPK_3338	YPK_3339	137, 198
IrGS37	3674947	4.2	5.4	3.6	ATGGAACAGGACTTGCAT	−	9.85	σ^24,54^	YPK_3349	NA	−42
IrGS38	3948038	5.0	3.0	2.3	CCGGCCCGCATGATGCCG	−	8.67	σ^54,38,24^	YPK_3583	NA	10, 38
IrGS39	4007370	3.1	NA	NA	TTGGTGCGGATTATGCAG	−	9.57	σ^38,24,54^	YPK_3630	YPK_3629	−30, −197
IrGS40	4106580	3.0	NA	2.5	CTGGCACCAGTTATGCGT	−	10.6	σ^24,38,32,54,28^	YPK_3718	YPK_3717	−47, −49, −50, 107
IrGS41	4186557	5.2	3.9	3.1	AAGGGGCAATCTTTGCAA	−	8.94	σ^38,54,24^	YPK_3799	YPK_3798	−134, 194
IrGS42	4216234	11.9	9.6	7.5	TTGGTATAGGTTTTGCAG	+	10.1	σ^32,54^	YPK_3826	YPK_3827, YPK_3828	−167
IrGS43	4341322	3.8	NA	NA	ATGGCACAGTTACTGCAG	+	7.68	NA	YPK_3934	NA	NA
IrGS44	4441905	10.2	14.2	9.2	TTGGCACGGAAAATGCTA	−	10.5	σ^24,32,54^	YPK_4027^S^	YPK_4026	−104, −137
IrGS45	4448335	8.6	11.2	7.7	TTGGCATTGTTCTTGCTT	−	11.1	σ^24,54^	YPK_4033	YPK_4032^E,S^	−148
IrGS46	4561060	5.1	2.7	3.2	TTGGTATGGTTTTCGCCG	−	9.67	σ^32,54,70^	YPK_4127	YPK_4126	−179, 123
IrGS47	4584577	4.5	4.3	3.3	TTGGTACGGGGTTTGCGT	+	10.1	σ^54,70,24^	YPK_4151	NA	133, 191
IrGS48	4602274	5.6	4.2	4.0	TTGGCGCACAAATTGCTC	+	10.1	NA	YPK_4174	NA	NA
IrGS49	4612122	5.6	4.2	4.0	ATGGCACGCTAGTTGCGG	−	10.6	σ^54,70,24^	YPK_4181^V^	NA	173, 123
Intragenic antisense (D sites)											
IrGAs1	128696	3.0	NA	NA	CTGGCCCAATACATGCAT	−	10.1	σ^54,28,24,32^	YPK_0107	YPK_105, YPK_106	2, 11, 27
IrGAs2	318299	NA	2.9	NA	CTGGCTTGTCGGATGCAC	−	9.06	σ^38,54,32^	YPK_0280	YPK_0289	−44, 132
IrGAs3	549836	4.7	3.3	NA	TTGGTCCAGTAATTGCTG	+	9.9	σ^54,32,38^	YPK_0496	YPK_0495	15, 164
IrGAs4	630656	3.3	2.6	NA	ACGGCTCAATTGTTGCAT	−	9.97	σ^24,54,32,28^	YPK_0712	YPK_0713, YPK_0714	−19, 20, 140
IrGAs5	1053922	3.6	NA	NA	CAGGCACGATACGTGCAT	+	10.1	σ^54,24,38^	YPK_0933		168, 172
IrGAs6	1099750	2.8	NA	NA	CCGGCATGCATCATGCAC	+	9.39	σ^24,54,38^	YPK_0978	YPK_0979	−8, 159
IrGAs7	1204307	2.5	NA	NA	TTGGTACAGTTTTTTCCA	−	9.45	σ^54,32,28^	YPK_1066	YPK_1065	109, 112
IrGAs8	1265403	12.0	7.4	8.0	TTGGCATGCATATTGCGC	−	10.9	σ^38,54,24,32,70^	YPK_1125	YPK_1126	−27, 4, 8, 21
IrGAs9	1357208	5.8	4.3	3.1	CTGGCATGTCATTTGCTG	−	10.6	σ^54,70,38,28^	YPK_1228	NA	63, 152, 182
IrGAs10	1894351	3.5	NA	NA	TGGGCACACTCGTTGCAT	−	10	σ^54,28,24,38^	YPK_1706	NA	42, 44, 167
IrGAs11	2058280	6.7	4.8	3.7	AATGCACGTTTCATGCTT	−	9.09	σ^54,28,32,38^	YPK_1853	NA	67, 152, 182
IrGAs12	2760688	8.9	6.0	3.7	TAGGGTTTCTGGCTGCGG	+	7.27	σ^32,24,54,28^	YPK_2519	YPK_2520	−39, −22, 128
IrGAs13	3195552	4.2	NA	3.5	TGGGCATAATTTTTGCCA	−	10.6	σ^54,28^	YPK_2901	NA	19
IrGAs14	3296918	10.0	8.8	7.9	TAGGCACGAGATTTGCAT	−	10.7	σ^54,32,28,38^	YPK_2997	YPK_2996^E^	168, 189, 199
IrGAs15	3347078	3.5	NA	NA	TTGGCAAGAAATCTGCAC	+	10.7	σ^38,70,54,28^	YPK_3045	NA	−5, −7, 3
IrGAs16	3383005	13.6	11.8	9.3	ATGGCACGGATTTTGCCC	−	10.8	σ^38,24,54,70^	YPK_3075	YPK_3074	−43, −50, 72
IrGAs17	3435112	5.4	3.7	NA	AAGGCACGATAGTTGCGT	−	10.5	σ^54,28,32,38^	YPK_3144	NA	52, 104, 119
IrGAs18	3603190	3.1	NA	NA	CTGGCACTGATATTGCCG	−	10.2	σ^70,32,54,38^	YPK_3293	NA	−26, −8, 187
IrGAs19	3621908	4.7	5.0	4.4	ATGGCATCAATATTGCTA	+	10.5	σ^54,32,28^	YPK_3308	YPK_3309	106, 113
IrGAs20	3984487	6.0	5.0	3.6	CTGGCTTAAATCTTGCGT	+	10.4	σ^54,24,38,32^	YPK_3613	YPK_3614	75, 110, 125
IrGAs21	4135862	4.4	NA	3.5	CTGGCACGCTTTCTGCAA	+	10.6	σ^32,54,24,38^	YPK_3743^E^	NA	−15, 22, 123
IrGAs22	4186557	5.2	3.9	3.1	CAGGAACGGATCTTGCAA	+	9.38	NA	YPK_3799	NA	
IrGAs23	4288435	11.2	13.7	10.2	CTGGCTCAATTAATGCAT	−	10.3	σ^24,28,54^	YPK_3887	NA	−102, −178
IrGAs24	4309656	5.1	5.0	3.4	CTGGTGTAAATATTGCAC	+	9.89	NA	YPK_3909	NA	
IrGAs25	4371420	11.5	7.9	5.2	GCGGCGCATTTTTTGCAT	+	9.83	σ^24,32,54^	YPK_3962	NA	−143, −198

a Abbreviations: IrGS, intragenic sense; IrGAs, intragenic antisense; PC, peak center base pair; PSL, peak strength logarithmic phase; PSI, peak strength induction; PSO, peak strength over expression of RpoN; PSSM, position-specific score matrix; X^E/S/V^, prefix indicating existence of RpoN binding motif in similar location in relation to orthologous genes in E. coli (E), V. cholerae (V), or *S*. Typhimurium (S); NA, not applicable.

10.1128/mSystems.01006-20.5 TABLE S2 Low-confidence peaks of RpoN binding site. Download Table S2, PDF file, 0.3 MB. Copyright © 2020 Mahmud et al. 2020 Mahmud et al. This content is distributed under the terms of the Creative Commons Attribution 4.0 International license.

The robustness of the analysis was further verified by the identification of RpoN binding sites at intergenic regions upstream of genes previously shown to be regulated by RpoN in other bacteria. Examples here are YPK_1894 (*pspA*), YPK_3220 (*glnK*), YPK_3857 (*pspG*), and YPK_4189 (*glnA*) (8, 27, 28). Comparing our high-stringency peaks with the peaks identified by ChIP-seq in E. coli and *S*. Typhimurium, it was obvious that the location of many of the peaks in relation to coding DNA sequences (CDS) was conserved, and some were also shared with V. cholerae (Fig. 2D and Tables 1, 2, and 3). The conserved locations of RpoN binding sites included locations upstream of *pspA* and *glnA* with orthologs in E. coli, *S*. Typhimurium, and V. cholerae, for example, YPK_2229, YPK_2908, and YPK_1600, with orthologs in E. coli and *S.* Typhimurium (Table 3). There were also many novel binding sites identified in Y. pseudotuberculosis, where a majority were intragenic with some also on the noncoding strand (Fig. 2E and F). The presence of sense and antisense intragenic binding sites for RpoN is in accordance with what has been previously found for *S*. Typhimurium and E. coli (27, 28). The function and mechanisms of RpoN intragenic binding are generally unknown, but there are examples where this binding can drive transcription of downstream genes with long 5′ untranslated regions (UTRs) (27, 34). The putative RpoN binding sites identified were divided into groups (A to D) based on their position and orientation. Groups A (26 sites) and B (3 sites) comprise binding sites in intergenic regions. Group A sites are oriented toward the 5′ end of the nearest coding sequence, and those in group B are oriented toward the 3′ end of the neighboring coding sequence (Fig. 2E and F; Table 1). Groups C (49 sites) and D (25 sites) comprise intragenic binding sites; those in group C are oriented in the sense direction and those in group D in the antisense direction (Fig. 2E and F; Table 2). In general, binding sites in group A were associated with stronger peaks than the sites in groups C and D (Tables 1 and 2). Further, compared with the intergenic binding sites among which a relatively large fraction appears to be conserved among E. coli, *S*. Typhimurium, and Y. pseudotuberculosis, the fraction of conserved intragenic RpoN binding sites was considerably lower (Tables 1, 2, and 3).

**TABLE 3:** Existence of RpoN binding motif with similar location in relation to orthologous genes^*a*^

Peak	Locus tag	Description	Type	Y. pseudotuberculosis	E. coli	V. cholerae	*S*. Typhimurium
IGS8	YPK_1600	Glutamine ABC transporter periplasmic protein, GlnH	A	√	√		√
IGS10	YPK_1894	Phage shock protein, PspA	A	√	√	√	√
IGS14	YPK_2229	Bifunctional succinylornithine transaminase	A	√	√		√
IGS17	YPK_2908	Nitrogen specific histidine kinase, NtrB	A	√	√		√
IGS17	YPK_2909	Zinc resistance protein	A	√	√		√
IGS21	YPK_3220	Nitrogen regulatory protein P-II 2	A	√	√		√
IGS24	YPK_3857	Phage shock protein G, PspG	A	√	√		√
IGS26	YPK_4189	Glutamine synthetase, GlnA	A	√	√	√	√
IrGS45	YPK_4032	Lipopolysaccharide biosynthesis protein, WzzE	C	√	√		√

a Abbreviations and symbols: IGS, intergenic sense; IrGS, intragenic sense; locus tag, locus tag of the gene in relation to predicted RpoN binding site in Y. pseudotuberculosis YPIII; √, reported RpoN binding motif with similar location in relation to orthologous genes.

To reveal possible sigma factor cross talk in Y. pseudotuberculosis, we also screened for RpoD, RpoE, RpoS, RpoH, and FliA binding sites close (±200 nt) to the identified RpoN binding sites. This screen showed many potential dual and sometimes triple sigma factor binding regions close to each other in 68% of all A sites (Table 1). Even more multiple binding regions were found associated with intragenic sites, with clusters of 2 to 5 sigma factor sites in more than 90% of all C and D sites (Table 2). Notably, all sigma factor binding regions associated with A sites had a binding site for RpoD (σ^70^), and nearly half of them contained sites for RpoE (σ^24^) and/or RpoS (σ^38^). In contrast, binding sites for FliA (σ^28^) were very rare and sites for RpoH (σ^32^) were absent. The multiple binding sites associated with C sites included sites for RpoE (60%), RpoH (54%), RpoS (40%), FliA (42%), and RpoD (30%). D sites were similar, with binding sites for RpoS (64%), RpoH (60%), FliA (52%), RpoE (48%), and RpoD (20%).

### Deletion of *rpoN* has a large impact on the Y. pseudotuberculosis transcriptome, with both direct and indirect effects.

**FIG 3:**
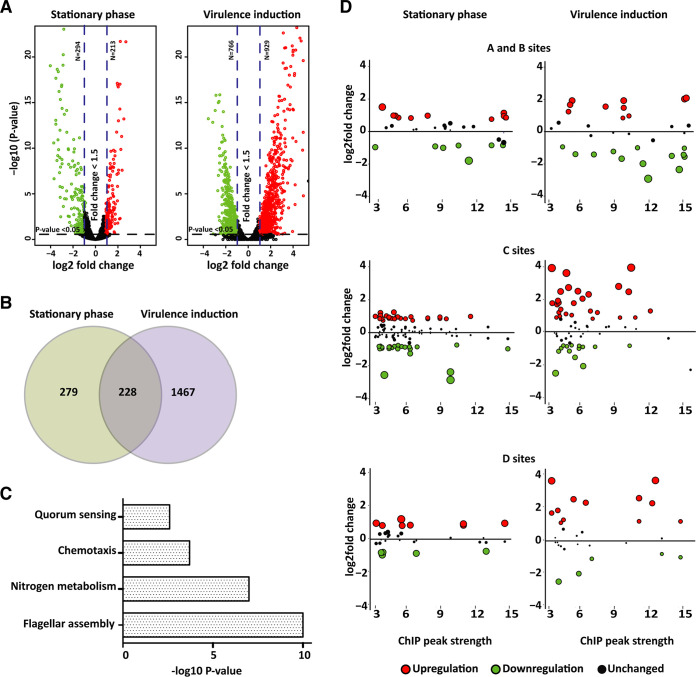
Deletion of *rpoN* has a substantial influence on the Y. pseudotuberculosis transcriptome. (A) Volcano plot showing differentially expressed genes in the Y. pseudotuberculosis Δ*rpoN* strain compared with the WT strain at stationary phase (OD = 2.0 at 26°C) and during virulence induction (Ca^2+^ depletion, at 37°C). Upregulated genes are indicated in red and downregulated genes in green. (B) Venn diagram showing the number of differentially expressed genes under the indicated conditions. The criteria for differential expression shown in panels A and B were fold changes of >1.5 with a *P* value of <0.05. (C) Pathway enrichment (KEGG) of genes differentially expressed both in stationary phase and after virulence induction. Pathways are ranked by the negative log_10_ of the *P* value of the enrichment score. The *P* values were calculated using the Bonferroni correction. (D) Plots showing expression of genes associated with intergenic or intragenic high-confidence peaks (A, B, C, and D sites). The *y* axis indicates expression values from RNA-seq at stationary phase and virulence induction (log_2_ fold change of Δ*rpoN*/WT), and the *x* axis indicates the strength of the associated ChIP peaks (the RpoN binding peak over control in log_2_ fold) where higher peak strength corresponds to stronger enrichment of RpoN within a binding site. Significantly upregulated (red circles) and downregulated (green circles) genes, as well as nonsignificant changes (black circles), are indicated for the different binding site groups. The portion of potential RpoN binding sites associated with differential gene expression was 88% for group A, 79% for group C, and 68% for group D.

Next, we aimed to determine whether the identified binding sites could indeed be coupled to gene expression in Y. pseudotuberculosis. For this we employed transcriptome sequencing (RNA-seq) on WT and Δ*rpoN* bacteria at 26°C in stationary phase and at 37°C with virulence induction. Analysis of differentially expressed genes revealed a markedly different gene expression pattern in the Δ*rpoN* strain compared with the isogenic WT strain. More than 500 genes were found to be differentially regulated at 26°C in stationary phase: 294 were downregulated and 213 were upregulated in Δ*rpoN* (Fig. 3A and B). The effect was even more pronounced at 37°C under virulence-inducing conditions: almost 1,700 genes were affected, with 766 genes downregulated and 929 upregulated. The reason for this discrepancy, with a much higher number of genes affected under virulence-inducing conditions compared with 26°C at stationary phase, might be a higher degree of stress associated with the former. This is a condition known to involve activation of different alternative sigma factors as well as other global regulators. Functional annotation analysis of the differentially expressed genes showed downregulation of genes involved in nitrogen metabolism, flagellar assembly, chemotaxis, and quorum sensing under both conditions (Fig. 3C and Fig. S2). There was also downregulation of fatty acid biosynthesis and metabolism, but this was not seen in samples of bacteria subjected to virulence-inducing conditions. For these samples, additional pathways were affected by the deletion of *rpoN*, including, for example, low expression of genes involved in the type III secretion system (T3SS) that normally is highly upregulated under this condition, downregulation of DNA replication and amino acid biosynthesis, and upregulation of genes involved in carbon metabolism, gluconeogenesis, and ribosomal organization, with the latter possibly reflecting the stress of translational reprogramming.

10.1128/mSystems.01006-20.2 FIG S2 Pathway enrichment in differentially expressed genes (Δ*rpoN*/WT) at stationary phase and under virulence induction. The pathway enrichment score was calculated as the probability of a gene’s enrichment in a pathway other than by random chance. Pathways are ranked by the negative log_10_ of the *P* value of the enrichment score. The *P* values were calculated using the Bonferroni correction and used to evaluate the significance of the pathway under a particular condition. Up- and downregulation of the pathway were predicted by calculating the Z score of differential expression of the genes in a certain pathway. Download FIG S2, PDF file, 0.1 MB. Copyright © 2020 Mahmud et al. 2020 Mahmud et al. This content is distributed under the terms of the Creative Commons Attribution 4.0 International license.

Although the expected effects of RpoN deletion, such as reduced expression of genes involved in nitrogen metabolism, flagella, and quorum sensing were obvious, the impact on the Y. pseudotuberculosis transcriptome was massive, clearly reflecting deletion of a global regulator. This accords with previous studies, which found that the absence of RpoN commonly results in a global effect involving both direct and indirect effects on gene expression (9, 24, 35). The differential gene expression analysis revealed changed expression levels of other sigma factors in the Δ*rpoN* strain (Table S3). Expression of RpoD, for example, was significantly upregulated, whereas expression of RpoE was downregulated both in stationary phase and under virulence-inducing conditions. RpoS, on the other hand, was upregulated in stationary phase but downregulated under inducing conditions. In addition to other sigma factors, the mRNA levels of other transcriptional regulators were affected, such as those of CpxR, RovA, FlhCD, and others (Table S3). Hence, these effects, together with effects on the transcription of other sigma factors, are expected to contribute extensively to indirect effects on gene expression, adding further complexity to the data set.

10.1128/mSystems.01006-20.6 TABLE S3 Global regulators differentially expressed in Δ*rpoN* strain. Download Table S3, PDF file, 0.4 MB. Copyright © 2020 Mahmud et al. 2020 Mahmud et al. This content is distributed under the terms of the Creative Commons Attribution 4.0 International license.

### Identified RpoN binding sites mediate both positive and negative regulation of gene expression.

Given the complexity, including indirect effects, in the RNA-seq data set, we next aimed to reveal direct effects of RpoN. The RpoN binding sites identified in the ChIP-seq analysis likely include both active promoters driving transcription and suppressive and silent binding of RpoN to the chromosome. The potential RpoN binding sites identified were therefore matched with detected changes in gene expression levels (Fig. 3D). Among the identified binding sites in group A, 88% were associated with differential gene expression, involving both upregulated and downregulated genes (Fig. 3D). The intragenic C and D sites were also associated with differential gene expression, 79% for the C sites and 68% for the D sites. For C sites, the differential gene expression included both upregulation and downregulation of genes containing the motif as well as downstream genes. For the antisense D sites, there was a larger fraction of upregulated than downregulated genes, suggesting potential suppressive effects of the RpoN binding (Fig. 3D). In general, a relatively large portion of the differentially expressed genes associated with RpoN binding showed increased expression in the Δ*rpoN* strain. Binding by RpoN might suppress transcription by nearby sigma factors and possibly other transcription factors binding to the same region, where the absence of RpoN would then allow transcription to occur (8, 27). Also, collision as a consequence of RpoN intragenic binding has been suggested (36, 37).

**FIG 4:**
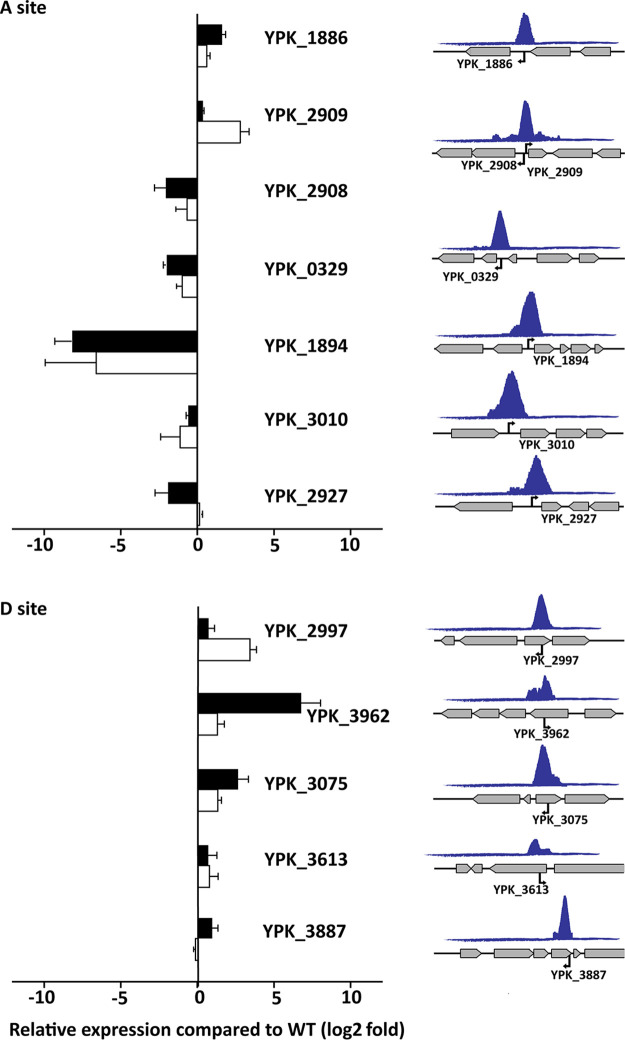
Validation of productive RpoN binding. (Left) Relative expression of indicated genes in bacteria when the associated RpoN binding site is mutated (white bars) and in the Δ*rpoN* strain (black bars). Expression of genes in the mutant strains was determined by qPCR and presented as log_2_ fold relative expression compared to expression in the WT strain, where positive values represent suppressed transcription and negative values indicate activated transcription. Results are shown for expression under virulence-inducing conditions for YPK_0329, YPK_1886, YPK_1894, YPK_2927, YPK_2997, YPK_3010, YPK_3075, YPK_3613, YPK_3887, and YPK_3962 and for expression during stationary phase at 26°C for YPK_2908 and YPK_2909. Values represent means ± SEM of three independent experiments. (Right) Schematic illustration of the shapes and genomic contexts of ChIP peaks together with the positions and orientations of RpoN binding of associated motifs.

To explore the potential importance of the RpoN binding sites identified in Y. pseudotuberculosis, we set out to mutate some of them to reveal their effects on gene expression. For this, we chose binding sites with locations indicative of putative positive or negative regulation by RpoN, which was the case for many A and D sites. We could not identify any C-site ChIP-seq peak indicative of transcriptional activation of downstream genes in our data set. The sites were mutated by the exchange of 3 to 6 nucleotides in the conserved TGG and TGC sequences of the RpoN binding motif. In intragenic motifs, the exchanged nucleotides were selected in order to minimize changes in the encoded protein (see Table S5 for details). The selected sites included seven A and five D sites (Fig. 4 and Table S4), and the effects of the mutations on gene expression were verified by qPCR. For all putative activating A sites, those represented by downstream genes downregulated in the absence of RpoN, point mutations in the RpoN binding motifs in the WT strain resulted in reduced transcription. This class of binding sites also showed the highest degree of conservation. The most prominent effect of the disruptive nucleotide exchange was seen for *pspA* (YPK_1894), a gene known to be activated by RpoN (38, 39). There were also indications of inhibitory effects of RpoN binding to A sites, where the expression of downstream genes was increased in both the Δ*rpoN* strain and the corresponding binding site mutant. Intriguingly, four of the five binding site mutations in D sites resulted in increased expression of the CDS on the opposite strand, suggesting a suppressive effect of RpoN binding. We also mutated some of the C sites, but here no effect on transcription compared with that in the WT strain could be seen. Notably, peaks associated with C sites were commonly flatter and broader than the peaks covering the mutated A and D binding sites (Fig. 4 and Fig. S3).

10.1128/mSystems.01006-20.7 TABLE S4 Validations of productive RpoN binding. Download Table S4, PDF file, 0.3 MB. Copyright © 2020 Mahmud et al. 2020 Mahmud et al. This content is distributed under the terms of the Creative Commons Attribution 4.0 International license.

10.1128/mSystems.01006-20.8 TABLE S5 List of strains used in this study. Download Table S5, PDF file, 0.6 MB. Copyright © 2020 Mahmud et al. 2020 Mahmud et al. This content is distributed under the terms of the Creative Commons Attribution 4.0 International license.

10.1128/mSystems.01006-20.3 FIG S3 Schematic representation of selected intragenic sense (C) RpoN binding sites for qPCR. The arrow indicates the position and direction of the RpoN binding motif. ChIP peaks are shown in blue, representing their coverage in the genome of logarithmically grown bacteria (26°C). Download FIG S3, PDF file, 0.4 MB. Copyright © 2020 Mahmud et al. 2020 Mahmud et al. This content is distributed under the terms of the Creative Commons Attribution 4.0 International license.

Taken together, we have by mutagenesis been able to verify effects of RpoN binding to the binding motifs identified in a ChIP-seq screen of Y. pseudotuberculosis. Activating as well as suppressing effects of intergenic RpoN binding were verified by mutating intergenic A sites. Among these verified productive RpoN binding sites, some were known, such as those in PspA, PspP, and GlnA, whereas RpoN regulation of TppB, UgpB, and DkgA is described for the first time. There were also indications of inhibitory effects of RpoN binding to intragenic D sites. As discussed earlier, how RpoN suppresses transcription is less clear, but for binding to the sense strand it might occur by steric hindrance, either by the RpoN-RNA polymerase complex or by RpoN alone binding to the DNA. How this can affect expression from the opposite strand, as would be the case for the observed inhibitory effect of mutating RpoN binding D sites, is less clear, but it might involve disturbed strand separation. For intragenic RpoN binding, we saw effects on transcription only by mutating binding sites on the antisense strand. Thus, intragenic binding by RpoN to coding regions on the sense strand is likely silent and may be used for storage of RpoN-RNAP. RpoN-mediated suppression by binding to internal antisense sequences of genes carried on the opposite strand has not been shown previously, and its mechanism remains to be elucidated.

## MATERIALS AND METHODS

### Bacterial strains and growth conditions.

Strains and plasmids are listed in Table S5 in the supplemental material. Yersinia pseudotuberculosis strain YPIII was used in this study. Escherichia coli S17-1 λpir was used for cloning and conjugation. Antibiotics were used at the following concentrations: ampicillin (100 μg/ml), kanamycin (50 μg/ml), and chloramphenicol (25 μg/ml). Motility was tested on Luria-Bertani (LB) medium with 0.6% agar. Biofilm assays were carried out as described previously, using LB medium in glass tubes (40). All strains were routinely grown at 26°C in LB medium containing kanamycin (50 μg/ml). For ChIP- and RNA-seq analyses, cultures were grown in LB medium to the desired OD_600_. Arabinose (0.005%) was used for 30-min induction at 26°C for overexpression of *rpoN*. To reach the virulence induction condition, overnight bacterial cultures were diluted to an OD_600_ of 0.05 in LB and grown at 26°C. After 1 h, calcium was depleted by adding 5 mM EGTA and 20 mM MgCl_2_ and cultures were shifted to 37°C (22).

### Strain construction.

In-frame gene deletion and insertion of the V5 epitope and binding-site mutations in Y. pseudotuberculosis were performed using an In-Fusion HD cloning kit (Clontech) according to the manufacturer’s instructions. Briefly, the flanking regions of the respective gene were amplified by PCR and cloned into the suicide vector pDM4. This construct was used to transform S17-1 and then transferred into recipient strains through conjugation. Conjugants were purified and incubated on 5% sucrose to recombine out the vector together with WT sequence. Deletion or mutation was confirmed by PCR. For *trans*-complementation, the gene was PCR amplified and cloned into the pBAD24 plasmid. For gene induction, *rpoN* and a C-terminal 3×V5 epitope tag were PCR amplified and cloned into the pBAD18 plasmid. All constructs were verified by sequencing. Primers used in this study are listed in Table S6.

10.1128/mSystems.01006-20.9 TABLE S6 Primers used in this study. Download Table S6, PDF file, 0.5 MB. Copyright © 2020 Mahmud et al. 2020 Mahmud et al. This content is distributed under the terms of the Creative Commons Attribution 4.0 International license.

### Ethics statement.

Mice were housed and treated in accordance with the Swedish National Board for Laboratory Animals guidelines. All the animal procedures were approved by the Animal Ethics Committee of Umeå University (Dnr A108-10). Mice were allowed for 1 week to conform to the new environment before the experiments started.

### Mouse infections.

Female FVB/N mice (Taconic) 8 weeks old were deprived of food and water for 16 h before infection. For infection of mice, overnight cultures of the Y. pseudotuberculosis strains were suspended in sterilized tap water supplemented with 150 mM NaCl, reaching an approximate CFU count of 10^6^/ml for low-dose infection and 10^8^ CFU/ml for acute infection. Mice were allowed to drink for 6 h. The infection dose was calculated based on viable count and the volume of drinking water supplemented with bacteria that was consumed. Frequent inspections of mice were carried out routinely to ensure no prominent clinical signs were overlooked. Infected mice showing notable clinical signs were euthanized promptly to prevent suffering.

The infections were monitored using the IVIS Spectrum *in vivo* imaging system (Caliper Life Sciences) routinely every 3rd day until 15 days postinfection (dpi) and later every week up to 28 dpi. The mice were anesthetized using the XGI-8 gas anesthesia system (Caliper Life Sciences) and 2.5% IsoFloVet (Orion Pharma, Abbott Laboratories Ltd., Great Britain) in oxygen for initial anesthesia and 0.5% isoflurane in oxygen during IVIS imaging. After infection, some mice were euthanized and dissected to analyze bacterial localization and presence in various organs, including the intestine, mesenteric lymph nodes, liver, and spleen. The organs were imaged using IVIS. Living Image software, version 3.1 (Caliper Life Sciences, Inc.), was used for image acquisition and data analysis.

### Motility and biofilm assays.

Determination of swimming motility was performed as described previously (41). A 5-μl aliquot of a diluted overnight culture (OD_600_ of 1.0) was spotted at the center of a 0.6% LB soft agar plate and incubated at 26°C for 24 h. Bacterial motility was determined by measuring the diameter of the bacterial growth area.

Biofilm formation was determined as previously described (40). Overnight cultures were diluted to an OD_600_ of 0.05 and grown to an OD_600_ of 0.5 in a 26°C shaking water bath. A 1-ml aliquot of the bacterial culture was pelleted and dissolved in 2 ml fresh LB. The suspension was transferred to glass tubes and incubated for 48 h at 37°C without shaking. After incubation, the bacterial suspension was discarded and tubes were gently washed 3 times with PBS and stained with 0.1% crystal violet (Sigma-Aldrich) for 15 min, followed by 3 successive washes with PBS. The biofilms on the tube surface were thereafter dissolved with 33% acetic acid for 15 min, and the absorbance at 590 nm was measured with an Ultrospec 2100 Pro spectrophotometer (Amersham Biosciences, Piscataway, NJ).

### Chromatin immunoprecipitation followed by next-generation sequencing (ChIP-seq).

Y. pseudotuberculosis Δ*rpoN* expressing RpoN::V5 in *trans* under the inducible araBAD promoter (YPIII, Δ*rpoN*/pIBX, p*rpoN*::V5) and Y. pseudotuberculosis expressing RpoN:V5 in *cis* under its native promoter (YPIII, *rpoN*::V5/pIBX) were grown overnight in LB, diluted to an OD_600_ of 0.05, and grown for 4 h at 26°C, at which point the p*rpoN*::V5 plasmid was induced with 0.005% arabinose for 30 min. Virulence-induced cultures were shifted to 37°C after 1 h at 26°C and incubated for 75 min under calcium-depleted conditions. Nontagged WT Y. pseudotuberculosis grown at 26°C for 4 h was used as a negative control. Cultures were cross-linked with 1% formaldehyde at room temperature for 10 min. For subsequent ChIP-seq, 10 OD_600_ units was used for each biological replicate. Chromatin immunoprecipitation was performed as described previously, with slight modifications (27). The 1-ml samples were sonicated in AFA milliTUBEs using a Covaris E220 sonicator with the following settings: peak power, 140 W; duty factor, −5%; cycles/burst, 200; time, 16 min. Immunoprecipitation was done using 90 μl anti-V5 agarose affinity gel (Sigma A 7345) overnight at 4°C. ChIP DNA was eluted in 100 μl elution buffer, treated with RNase A and proteinase K, and purified using a ChIP DNA Clean & Concentrator kit (Zymo Research, USA). AMPure XP magnetic beads (Beckman Coulter), 1.5× and 0.8×, were used to remove adapters and clean and concentrate the DNA. Failsafe polymerase (Nordic Biolabs AB, Sweden) was used for efficient library amplification.

### ChIP-seq data analysis.

A custom pipeline in Python was used for ChIP-seq data analysis, data visualization, and downstream bioinformatic and computational analyses (the pipeline is available on request). The quality of raw reads generated from HiSeq 2500 was checked using FastQC (42). Raw reads were trimmed by 5 nt at both the 5′ and 3′ ends and aligned with the reference Y. pseudotuberculosis YPIII chromosome (NC_010465) and plasmid (NC_006153) using BWA (43). Aligned reads were converted to BAM files using SAMtools (44), and duplicate reads were removed with Picard (45) by deduplicate function. Peaks and RpoN binding sites are calculated in all three ChIP-seq samples with a stringent cutoff of false-discovery rate (FDR) (1E^−30^). Before peak calling, two biological replicates for each sample and the input (control) were merged using SAMtools. Peak calling was done using MACS (2.1.2) (46) with the following custom settings: log2FC > 1 over input, –broad –g, –broad-cutoff = 0.1. Identified peak coordinates in the genome were used to identify the probable regulated genes using the R packages ChIPpeakAnno and ChIPseeker (47, 48). *De novo* motif prediction was done using the bioconductor package BCRANK (49). A position-weight matrix (PWM) of the predicted motif was used to map the motif presence around the peak center using the FIMO-meme package (Linux version) (50). A region of 50 bases upstream and downstream of the peak center was used as input for searching for the PWM of the motif. The SIGffRid tool was used to predict the binding sites of other sigma factors within 200 bases upstream and downstream of each predicted RpoN binding peak center. To check the conservation of RpoN-regulated genes, cross-species homology prediction was done by comparing Y. pseudotuberculosis YPIII, V. cholerae 037, E. coli O157, and *S*. Typhimurium LT2 using OrthologueDB (51). *Yersinia* genes having orthologs in at least one of the species mentioned above were checked for previous ChIP-seq studies (24, 27, 28). Figures are plotted using the ggplot2 package in R (Linux version 4.0.2).

### RNA isolation and Illumina sequencing.

Total RNA was isolated from three independent biological replicates of WT Y. pseudotuberculosis and an isogenic *rpoN* deletion strain at stationary phase and under virulence-inducing conditions. For isolation and purification of RNA, the TRIzol method (Ambion Life Technologies, Carlsbad, CA) and the Direct-zol RNA kit (Zymo Research, USA) were used. To remove unwanted DNA, purified RNA was treated with DNase for 30 min at room temperature according to the manufacturer’s instructions. RNA quantity was measured by Qubit (Nordic Biolabs AB, Sweden). RNA from stationary-phase bacteria was collected after 8 h of growth (OD_600_ of ∼2) at 26°C in LB. For virulence-inducing conditions, RNA was isolated from cells grown exponentially at 26°C, shifted to 37°C, and incubated in Ca^2+^-depleted medium for 3 h.

For sequencing, cDNA libraries were prepared using the ScriptSeq Complete kit (Epicentre, Madison, WI, USA) according to the manufacturer’s instructions. rRNA was depleted from total RNA using a Ribo-Zero rRNA removal kit for bacteria (Epicentre, Madison, WI, USA) following the manufacturer’s protocol. The resulting cDNA libraries were purified using AMPure XP (Beckman Coulter) and quantified using an Agilent 2100 Bioanalyzer. Sequencing was done using an Illumina MiSeq.

Raw RNA-seq reads were trimmed from 5′ and 3′ ends by Trimmomatic (52) until all the adapter and low-quality bases (Phred quality score >30) were removed and the sequences passed quality checking by FastQC (42). ProkSeq, a complete RNA-seq data analysis package for prokaryotes, was used for further RNA-seq data processing, quality control, and visualization (53). It includes all the tools mentioned below as well as SAMtools (44) and BEDTools (54). Reads were aligned to the Y. pseudotuberculosis YPIII chromosome (NC_010465) and plasmid (NC_006153) using Bowtie 2 (55) with the unique-mapping option. Postmapped read quality checking was done using RSeQC (56), and the numbers of reads for each gene were counted using featureCounts (57). Differential gene expression was determined using DESeq2, using the shrinkage estimation of dispersion option (lfcSrink =True) to generate more accurate estimates of differential expression in fold changes (58). Differential expression of a gene was defined using absolute log_2_ fold change values of ≥1.5 and false-discovery rate values of <0.05. Figures are plotted using the ggplot2 package in R (Linux version 4.0.2) and GraphPad Prism (version 8.0).

### cDNA preparation and qPCR.

To validate the ChIP-seq and RNA-seq results, qPCR was performed using qPCRBio SyGreen Mix (PCR Biosystems) and a Bio-Rad CFX Maestro real-time PCR machine. Bacterial strains were grown for 2.5 h at 37°C under inducing conditions in LB (OD_600_ of ∼0.6) or at 26°C for 8 h, and total RNA was extracted as described above. Isolated RNA was used as the template for cDNA synthesis using a RevertAid First-Strand cDNA synthesis kit (Thermo Scientific). Experiments were done in triplicate for each mutant. Among 8 genes tested, YPK_0831 was the most stable under relevant conditions and was selected as an internal control in order to calculate the relative expression levels of tested genes, using appropriate primers (see Table S6 in the supplemental material).

### Data availability.

The RNA-seq and ChIP-seq data files have been deposited in Gene Expression Omnibus (GEO) under accession numbers GSE155606, GSE155607, and GSE155608. All the computer code and pipeline used in these studies are available on request.

## References

[1] Martinez-Bueno MA, Tobes R, Rey M, Ramos JL 2002 Detection of multiple extracytoplasmic function (ECF) sigma factors in the genome of Pseudomonas putida KT2440 and their counterparts in Pseudomonas aeruginosa PA01. Environ Microbiol 4:842–855. doi:10.1046/j.1462-2920.2002.00371.x. 12534467

[2] Avican K, Fahlgren A, Huss M, Heroven AK, Beckstette M, Dersch P, Fallman M 2015 Reprogramming of Yersinia from virulent to persistent mode revealed by complex in vivo RNA-seq analysis. PLoS Pathog 11:e1004600. doi:10.1371/journal.ppat.1004600. 25590628 PMC4295882

[3] deHaseth PL, Lohman TM, Burgess RR, Record MT Jr. 1978 Nonspecific interactions of Escherichia coli RNA polymerase with native and denatured DNA: differences in the binding behavior of core and holoenzyme. Biochemistry 17:1612–1622. doi:10.1021/bi00602a006. 350271

[4] Wosten MM 1998 Eubacterial sigma-factors. FEMS Microbiol Rev 22:127–150. doi:10.1016/S0168-6445(98)00011-4. 9818380

[5] Kazmierczak MJ, Wiedmann M, Boor KJ 2005 Alternative sigma factors and their roles in bacterial virulence. Microbiol Mol Biol Rev 69:527–543. doi:10.1128/MMBR.69.4.527-543.2005. 16339734 PMC1306804

[6] Osterberg S, del Peso-Santos T, Shingler V 2011 Regulation of alternative sigma factor use. Annu Rev Microbiol 65:37–55. doi:10.1146/annurev.micro.112408.134219. 21639785

[7] Hunt TP, Magasanik B 1985 Transcription of glnA by purified Escherichia coli components: core RNA polymerase and the products of glnF, glnG, and glnL. Proc Natl Acad Sci U S A 82:8453–8457. doi:10.1073/pnas.82.24.8453. 2867543 PMC390934

[8] Dong T, Yu R, Schellhorn H 2011 Antagonistic regulation of motility and transcriptome expression by RpoN and RpoS in Escherichia coli. Mol Microbiol 79:375–386. doi:10.1111/j.1365-2958.2010.07449.x. 21219458

[9] Lloyd MG, Lundgren BR, Hall CW, Gagnon LB, Mah TF, Moffat JF, Nomura CT 2017 Targeting the alternative sigma factor RpoN to combat virulence in Pseudomonas aeruginosa. Sci Rep 7:12615. doi:10.1038/s41598-017-12667-y. 28974743 PMC5626770

[10] Liu Y, Shi H, Wang Z, Huang X, Zhang X 2018 Pleiotropic control of antibiotic biosynthesis, flagellar operon expression, biofilm formation, and carbon source utilization by RpoN in Pseudomonas protegens H78. Appl Microbiol Biotechnol 102:9719–9730. doi:10.1007/s00253-018-9282-0. 30128583

[11] Matz C, Moreno AM, Alhede M, Manefield M, Hauser AR, Givskov M, Kjelleberg S 2008 Pseudomonas aeruginosa uses type III secretion system to kill biofilm-associated amoebae. ISME J 2:843–852. doi:10.1038/ismej.2008.47. 18480848 PMC2662702

[12] Ishikawa T, Rompikuntal PK, Lindmark B, Milton DL, Wai SN 2009 Quorum sensing regulation of the two hcp alleles in Vibrio cholerae O1 strains. PLoS One 4:e6734. doi:10.1371/journal.pone.0006734. 19701456 PMC2726435

[13] Suarez G, Sierra JC, Sha J, Wang S, Erova TE, Fadl AA, Foltz SM, Horneman AJ, Chopra AK 2008 Molecular characterization of a functional type VI secretion system from a clinical isolate of Aeromonas hydrophila. Microb Pathog 44:344–361. doi:10.1016/j.micpath.2007.10.005. 18037263 PMC2430056

[14] Hendrickson EL, Plotnikova J, Mahajan-Miklos S, Rahme LG, Ausubel FM 2001 Differential roles of the Pseudomonas aeruginosa PA14 rpoN gene in pathogenicity in plants, nematodes, insects, and mice. J Bacteriol 183:7126–7134. doi:10.1128/JB.183.24.7126-7134.2001. 11717271 PMC95561

[15] O’Toole R, Milton DL, Horstedt P, Wolf-Watz H 1997 RpoN of the fish pathogen Vibrio (Listonella) anguillarum is essential for flagellum production and virulence by the water-borne but not intraperitoneal route of inoculation. Microbiology 143:3849–3859. doi:10.1099/00221287-143-12-3849. 9421909

[16] Bush M, Dixon R 2012 The role of bacterial enhancer binding proteins as specialized activators of sigma54-dependent transcription. Microbiol Mol Biol Rev 76:497–529. doi:10.1128/MMBR.00006-12. 22933558 PMC3429621

[17] Hendrickson EL, Guevera P, Penaloza-Vazquez A, Shao J, Bender C, Ausubel FM 2000 Virulence of the phytopathogen Pseudomonas syringae pv. maculicola is rpoN dependent. J Bacteriol 182:3498–3507. doi:10.1128/jb.182.12.3498-3507.2000. 10852883 PMC101941

[18] Alarcon-Chaidez FJ, Keith L, Zhao Y, Bender CL 2003 RpoN (sigma(54)) is required for plasmid-encoded coronatine biosynthesis in Pseudomonas syringae. Plasmid 49:106–117. doi:10.1016/s0147-619x(02)00155-5. 12726764

[19] Schulz S, Eckweiler D, Bielecka A, Nicolai T, Franke R, Dotsch A, Hornischer K, Bruchmann S, Duvel J, Haussler S 2015 Elucidation of sigma factor-associated networks in Pseudomonas aeruginosa reveals a modular architecture with limited and function-specific crosstalk. PLoS Pathog 11:e1004744. doi:10.1371/journal.ppat.1004744. 25780925 PMC4362757

[20] Iyer VS, Hancock LE 2012 Deletion of sigma(54) (rpoN) alters the rate of autolysis and biofilm formation in Enterococcus faecalis. J Bacteriol 194:368–375. doi:10.1128/JB.06046-11. 22081387 PMC3256635

[21] Wolfe AJ, Millikan DS, Campbell JM, Visick KL 2004 Vibrio fischeri sigma54 controls motility, biofilm formation, luminescence, and colonization. Appl Environ Microbiol 70:2520–2524. doi:10.1128/aem.70.4.2520-2524.2004. 15066853 PMC383144

[22] Rosqvist R, Magnusson KE, Wolf-Watz H 1994 Target cell contact triggers expression and polarized transfer of Yersinia YopE cytotoxin into mammalian cells. EMBO J 13:964–972. doi:10.1002/j.1460-2075.1994.tb06341.x. 8112310 PMC394898

[23] Fahlgren A, Avican K, Westermark L, Nordfelth R, Fallman M 2014 Colonization of cecum is important for development of persistent infection by Yersinia pseudotuberculosis. Infect Immun 82:3471–3482. doi:10.1128/IAI.01793-14. 24891107 PMC4136198

[24] Dong TG, Mekalanos JJ 2012 Characterization of the RpoN regulon reveals differential regulation of T6SS and new flagellar operons in Vibrio cholerae O37 strain V52. Nucleic Acids Res 40:7766–7775. doi:10.1093/nar/gks567. 22723378 PMC3439928

[25] Dame RT, Rashid FM, Grainger DC 2020 Chromosome organization in bacteria: mechanistic insights into genome structure and function. Nat Rev Genet 21:227–242. doi:10.1038/s41576-019-0185-4. 31767998

[26] Meyer AS, Grainger DC 2013 The Escherichia coli nucleoid in stationary phase. Adv Appl Microbiol 83:69–86. doi:10.1016/B978-0-12-407678-5.00002-7. 23651594

[27] Bonocora RP, Smith C, Lapierre P, Wade JT 2015 Genome-scale mapping of Escherichia coli sigma54 reveals widespread, conserved intragenic binding. PLoS Genet 11:e1005552. doi:10.1371/journal.pgen.1005552. 26425847 PMC4591121

[28] Samuels DJ, Frye JG, Porwollik S, McClelland M, Mrazek J, Hoover TR, Karls AC 2013 Use of a promiscuous, constitutively-active bacterial enhancer-binding protein to define the sigma(5)(4) (RpoN) regulon of Salmonella Typhimurium LT2. BMC Genomics 14:602. doi:10.1186/1471-2164-14-602. 24007446 PMC3844500

[29] Barrios H, Valderrama B, Morett E 1999 Compilation and analysis of sigma(54)-dependent promoter sequences. Nucleic Acids Res 27:4305–4313. doi:10.1093/nar/27.22.4305. 10536136 PMC148710

[30] Bono AC, Hartman CE, Solaimanpour S, Tong H, Porwollik S, McClelland M, Frye JG, Mrazek J, Karls AC 2017 Novel DNA binding and regulatory activities for sigma(54) (RpoN) in Salmonella enterica serovar Typhimurium 14028s. J Bacteriol 199:e00816-16. doi:10.1128/JB.00816-16. 28373272 PMC5446619

[31] Teytelman L, Thurtle DM, Rine J, van Oudenaarden A 2013 Highly expressed loci are vulnerable to misleading ChIP localization of multiple unrelated proteins. Proc Natl Acad Sci U S A 110:18602–18607. doi:10.1073/pnas.1316064110. 24173036 PMC3831989

[32] Jain D, Baldi S, Zabel A, Straub T, Becker PB 2015 Active promoters give rise to false positive ‘Phantom Peaks’ in ChIP-seq experiments. Nucleic Acids Res 43:6959–6968. doi:10.1093/nar/gkv637. 26117547 PMC4538825

[33] Merrick M, Gibbins J, Toukdarian A 1987 The nucleotide sequence of the sigma factor gene ntrA (rpoN) of Azotobacter vinelandii: analysis of conserved sequences in NtrA proteins. Mol Gen Genet 210:323–330. doi:10.1007/BF00325701. 3481423

[34] Brown DR, Barton G, Pan Z, Buck M, Wigneshweraraj S 2014 Nitrogen stress response and stringent response are coupled in Escherichia coli. Nat Commun 5:4115. doi:10.1038/ncomms5115. 24947454 PMC4066584

[35] Kazakov AE, Rajeev L, Chen A, Luning EG, Dubchak I, Mukhopadhyay A, Novichkov PS 2015 Sigma54-dependent regulome in Desulfovibrio vulgaris Hildenborough. BMC Genomics 16:919. doi:10.1186/s12864-015-2176-y. 26555820 PMC4641369

[36] Lyubetsky VA, Zverkov OA, Rubanov LI, Seliverstov AV 2011 Modeling RNA polymerase competition: the effect of sigma-subunit knockout and heat shock on gene transcription level. Biol Direct 6:3. doi:10.1186/1745-6150-6-3. 21255416 PMC3038987

[37] Mauri M, Klumpp S 2014 A model for sigma factor competition in bacterial cells. PLoS Comput Biol 10:e1003845. doi:10.1371/journal.pcbi.1003845. 25299042 PMC4191881

[38] Weiner L, Brissette JL, Model P 1991 Stress-induced expression of the Escherichia coli phage shock protein operon is dependent on sigma 54 and modulated by positive and negative feedback mechanisms. Genes Dev 5:1912–1923. doi:10.1101/gad.5.10.1912. 1717346

[39] Maxson ME, Darwin AJ 2006 Multiple promoters control expression of the Yersinia enterocolitica phage-shock-protein A (pspA) operon. Microbiology (Reading) 152:1001–1010. doi:10.1099/mic.0.28714-0. 16549664 PMC1550779

[40] Merritt JH, Kadouri DE, O’Toole GA 2005 Growing and analyzing static biofilms. Curr Protoc Microbiol Chapter 1:Unit 1B.1. doi:10.1002/9780471729259.mc01b01s00. PMC4568995 18770545

[41] Morales-Soto N, Anyan ME, Mattingly AE, Madukoma CS, Harvey CW, Alber M, Deziel E, Kearns DB, Shrout JD 2015 Preparation, imaging, and quantification of bacterial surface motility assays. J Vis Exp (98):52338. doi:10.3791/52338. PMC4541456 25938934

[42] Andrews S 2010 FastQC: a quality control tool for high throughput sequence data. http://www.bioinformatics.babraham.ac.uk/projects/fastqc.

[43] Li H, Durbin R 2009 Fast and accurate short read alignment with Burrows-Wheeler transform. Bioinformatics 25:1754–1760. doi:10.1093/bioinformatics/btp324. 19451168 PMC2705234

[44] Li H, Handsaker B, Wysoker A, Fennell T, Ruan J, Homer N, Marth G, Abecasis G, Durbin R, 1000 Genome Project Data Processing Subgroup. 2009 The Sequence Alignment/Map format and SAMtools. Bioinformatics 25:2078–2079. doi:10.1093/bioinformatics/btp352. 19505943 PMC2723002

[45] Broad Institute. 2019 Picard. Broad Institute, Cambridge, MA.

[46] Zhang Y, Liu T, Meyer CA, Eeckhoute J, Johnson DS, Bernstein BE, Nusbaum C, Myers RM, Brown M, Li W, Liu XS 2008 Model-based analysis of ChIP-Seq (MACS). Genome Biol 9:R137. doi:10.1186/gb-2008-9-9-r137. 18798982 PMC2592715

[47] Zhu LJ, Gazin C, Lawson ND, Pages H, Lin SM, Lapointe DS, Green MR 2010 ChIPpeakAnno: a Bioconductor package to annotate ChIP-seq and ChIP-chip data. BMC Bioinformatics 11:237. doi:10.1186/1471-2105-11-237. 20459804 PMC3098059

[48] Yu G, Wang LG, He QY 2015 ChIPseeker: an R/Bioconductor package for ChIP peak annotation, comparison and visualization. Bioinformatics 31:2382–2383. doi:10.1093/bioinformatics/btv145. 25765347

[49] Ameur A 2019 BCRANK: predicting binding site consensus from ranked DNA sequences.

[50] Grant CE, Bailey TL, Noble WS 2011 FIMO: scanning for occurrences of a given motif. Bioinformatics 27:1017–1018. doi:10.1093/bioinformatics/btr064. 21330290 PMC3065696

[51] Fulton DL, Li YY, Laird MR, Horsman BG, Roche FM, Brinkman FS 2006 Improving the specificity of high-throughput ortholog prediction. BMC Bioinformatics 7:270. doi:10.1186/1471-2105-7-270. 16729895 PMC1524997

[52] Bolger AM, Lohse M, Usadel B 2014 Trimmomatic: a flexible trimmer for Illumina sequence data. Bioinformatics 30:2114–2120. doi:10.1093/bioinformatics/btu170. 24695404 PMC4103590

[53] Firoj Mahmud AKM, Nandi S, Fällman M 2020 ProkSeq for complete analysis of RNA-seq data from prokaryotes. bioRxiv doi:10.1101/2020.06.09.135822. PMC8034529 33367516

[54] Quinlan AR, Hall IM 2010 BEDTools: a flexible suite of utilities for comparing genomic features. Bioinformatics 26:841–842. doi:10.1093/bioinformatics/btq033. 20110278 PMC2832824

[55] Langmead B, Salzberg SL 2012 Fast gapped-read alignment with Bowtie 2. Nat Methods 9:357–359. doi:10.1038/nmeth.1923. 22388286 PMC3322381

[56] Wang L, Wang S, Li W 2012 RSeQC: quality control of RNA-seq experiments. Bioinformatics 28:2184–2185. doi:10.1093/bioinformatics/bts356. 22743226

[57] Liao Y, Smyth GK, Shi W 2014 featureCounts: an efficient general purpose program for assigning sequence reads to genomic features. Bioinformatics 30:923–930. doi:10.1093/bioinformatics/btt656. 24227677

[58] Love MI, Huber W, Anders S 2014 Moderated estimation of fold change and dispersion for RNA-seq data with DESeq2. Genome Biol 15:550. doi:10.1186/s13059-014-0550-8. 25516281 PMC4302049

