# Supplementary figures and images for: Genome-Scale Mapping Reveals Complex Regulatory Activities of RpoN in Yersinia pseudotuberculosis
Source: mSystems. 2020 Nov 10;5(6):e01006-20. doi: 10.1128/mSystems.01006-20 (PMC7657599; doi:10.1128/mSystems.01006-20)

Figure S1:

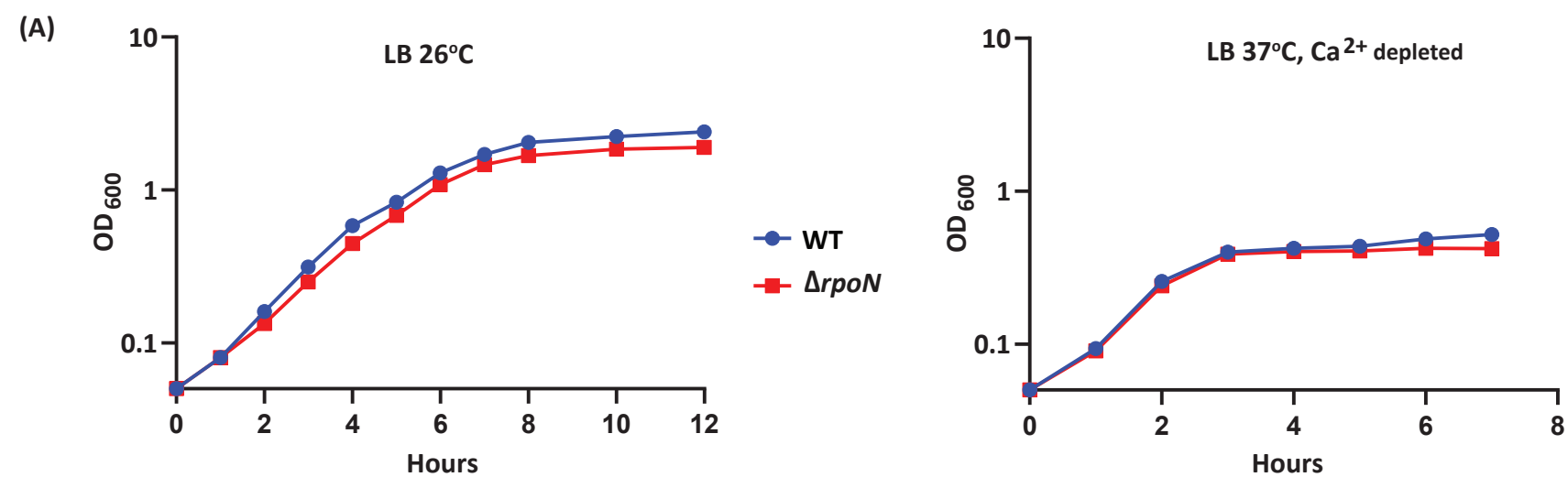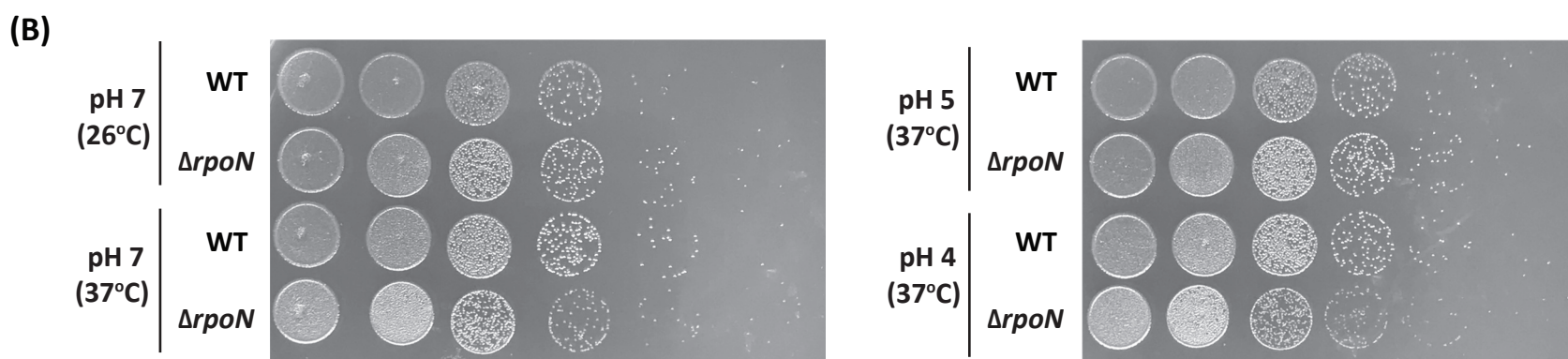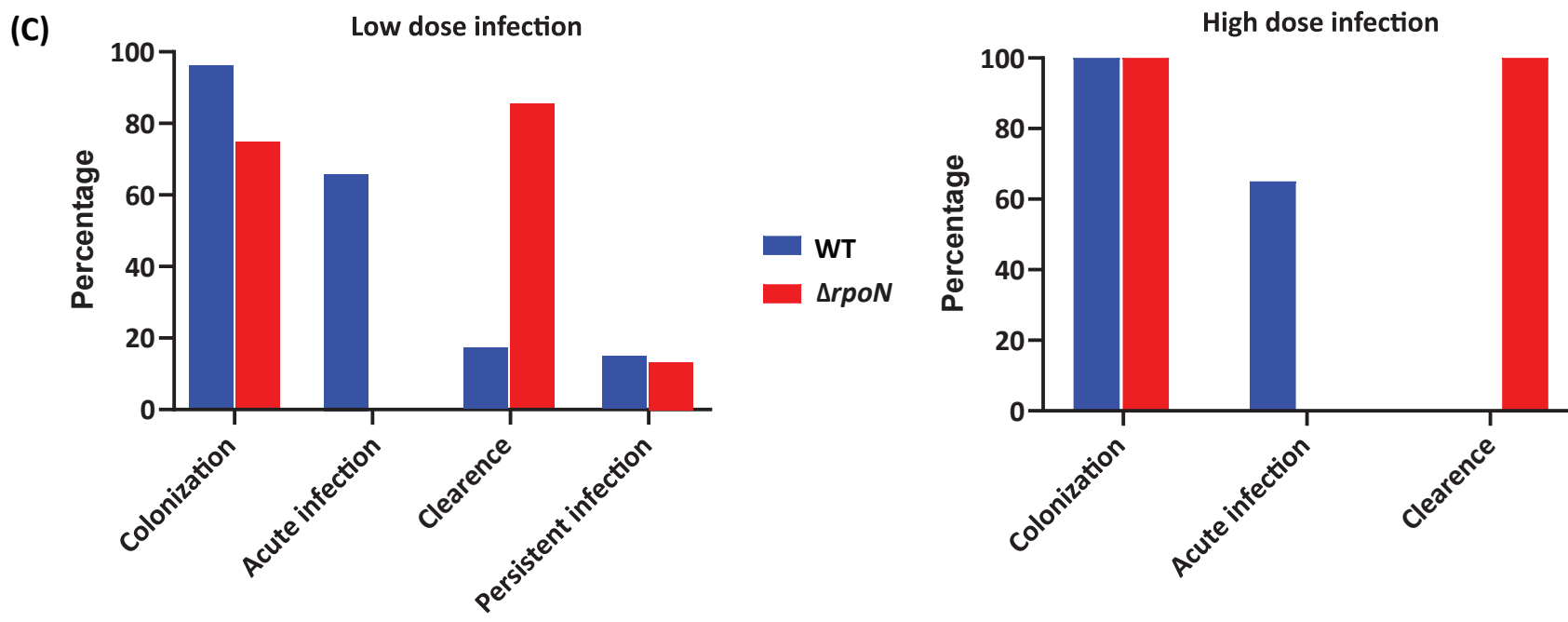

Supplement: FIG S1 [file mSystems.01006-20-sf001.pdf]

Figure S2:

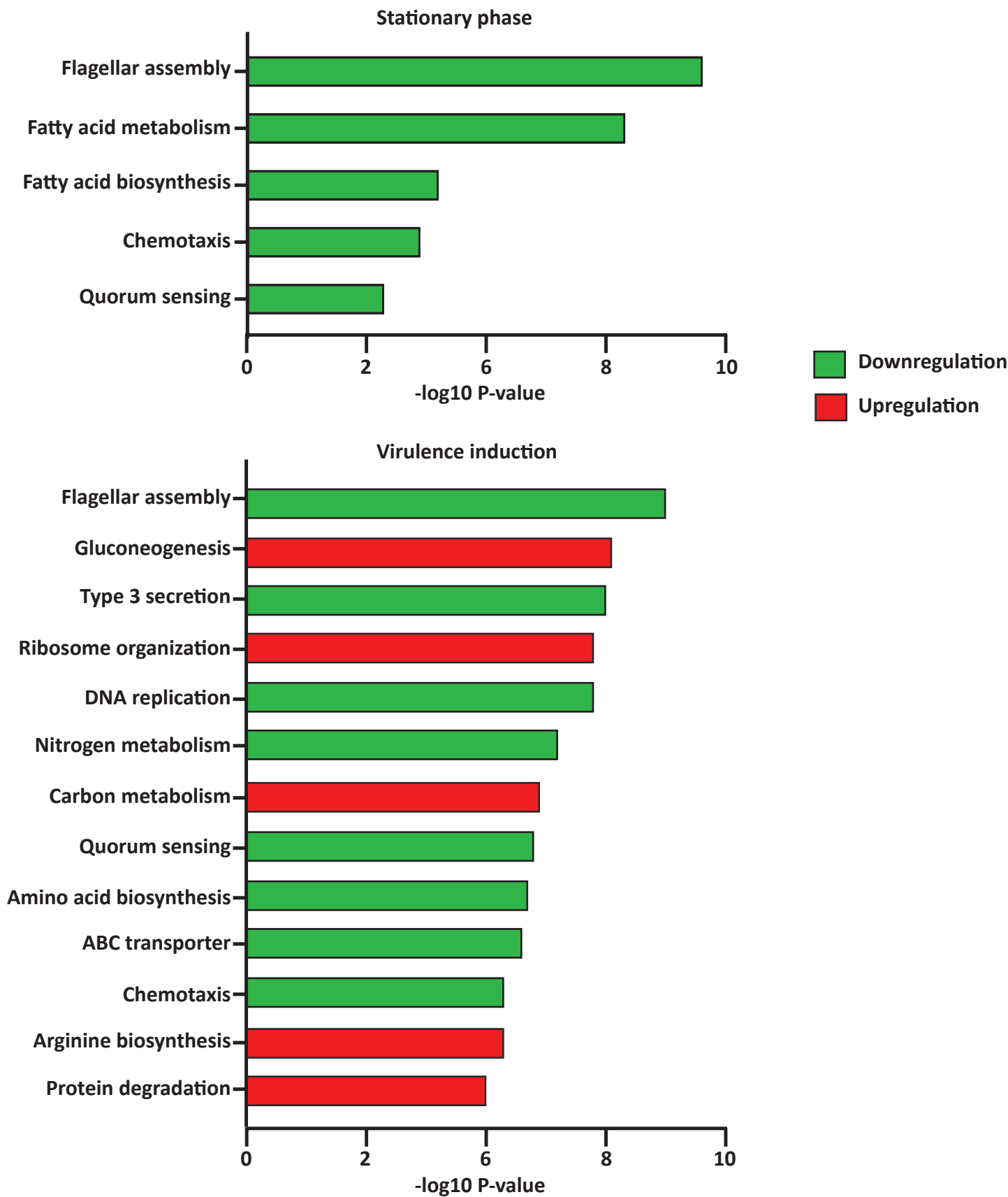

Supplement: FIG S2 [file mSystems.01006-20-sf002.pdf]

**Figure S3:**

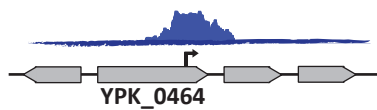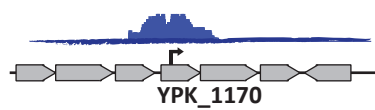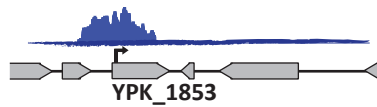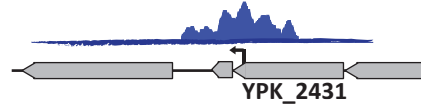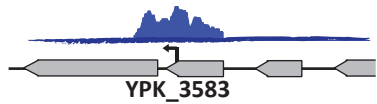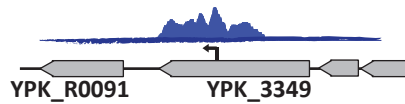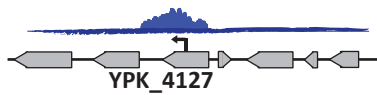

Supplement: FIG S3 [file mSystems.01006-20-sf003.pdf]
