# Supplementary material for: Genome-Scale Mapping Reveals Complex Regulatory Activities of RpoN in Yersinia pseudotuberculosis
Source: mSystems. 2020 Nov 10;5(6):e01006-20. doi: 10.1128/mSystems.01006-20 (PMC7657599; doi:10.1128/mSystems.01006-20)
Supplement: TABLE S1 [file mSystems.01006-20-st001.pdf]

Table S1: ChIP-seq peaks and predicted motif coordinates

Intergenic sense (A sites)

Peak	PC	PS	PE	PL	MS	ME	Strand(+/-)
IGS1	117503	117335	117566	231	117480	117497	+
IGS2	353423	353215	353602	387	353428	353445	-
IGS3	779812	779566	779995	429	779821	779838	+
IGS4	1127431	1127196	1127763	567	1127446	1127463	+
IGS5	1384233	1383971	1384417	446	1384210	1384227	+
IGS6	1469648	1469521	1469812	291	1469643	1469660	-
IGS7	1700633	1700542	1700877	335	1700637	1700654	+
IGS8	1770585	1770405	1770777	372	1770580	1770597	-
IGS9	2092867	2092677	2093076	399	2092869	2092886	-
IGS10	2101665	2101794	2102073	279	2101749	2101766	+
IGS11	2153679	2153542	2153837	295	2153683	2153700	-
IGS12	2194598	2194414	2194700	286	2194593	2194610	-
IGS12	2194598	2194414	2194700	286	2194695	2194714	+
IGS13	2298808	2298678	2299079	401	2298813	2298830	-
IGS13	2298808	2298678	2299079	401	2298793	2298810	-
IGS14	2473234	2473114	2473404	290	2473214	2473231	-
IGS15	2990747	2990598	2990815	217	2990727	2990744	+
IGS16	3172389	3171998	3172733	735	3172401	3172418	-
IGS17	3205379	3205232	3205497	265	3205374	3205391	-
IGS17	3205379	3205232	3205497	265	3205398	3205414	+
IGS18	3225401	3225139	3225654	515	3225404	3225421	+
IGS19	3300593	3300361	3300980	619	3300584	3300601	+
IGS20	3311541	3311362	3311769	407	3311529	3311546	+
IGS20	3311541	3311362	3311769	407	3311685	3311702	+
IGS20	3311541	3311362	3311769	407	3311497	3311514	+
IGS21	3520103	3519845	3520476	631	3520112	3520129	-
IGS22	3650370	3650106	3650598	492	3650369	3650386	-
IGS23	3651607	3651357	3651799	442	3651599	3651616	-
IGS23	3651607	3651357	3651799	442	3651598	3651615	+
IGS24	4250134	4249901	4250385	484	4250138	4250155	-
IGS25	4358596	4358227	4358884	657	4358592	4358609	-
IGS25	4358596	4358227	4358884	657	4358590	4358608	+
IGS26	4621308	4620967	4621427	460	4621215	4621232	-
IGS26	4621308	4620967	4621427	460	4621315	4621332	+

Intergenic antisense (B sites)

Peaks	PC	PS	PE	PL	MS	ME	Strand(+/-)
IGAs1	2892926	2892865	2893116	252	2892934	2892951	-
IGAs2	3172389	3172124	3172631	507	3172260	3172277	+
IGAs3	4432739	4432505	4432875	370	4432747	4432764	-

Intragenic Sense (C sites)

Peak	PC	PS	PE	PL	MS	ME	Strand(+/-)
IrGS1	173082	172873	173404	531	173107	173124	+

IrGS2	506362	506124	506627	503	506637	506649	+
IrGS3	535257	534929	535603	674	535265	535282	-
IrGS4	613541	613373	613616	243	613544	613561	-
IrGS5	653552	653206	653866	660	653539	653556	+
IrGS6	818023	817692	818400	708	818022	818039	-
IrGS7	821601	821323	821883	560	818022	818039	-
IrGS8	940388	940271	940540	269	940374	940391	+
IrGS9	1141660	1141501	1141837	336	1141669	1141686	+
IrGS10	1225391	1225255	1225702	447	1225385	1225402	+
IrGS11	1308478	1308280	1308833	553	1308497	1308514	+
IrGS12	1475860	1475786	1476016	230	1475837	1475854	+
IrGS13	1528613	1528276	1528948	672	1528595	1528612	+
IrGS14	1605153	1604808	1605506	698	1605150	1605167	-
IrGS15	1930700	1930416	1930974	558	1930704	1930721	-
IrGS16	2058280	2057954	2058596	642	2058240	2058257	+
IrGS16	2058280	2057954	2058596	642	2058239	2058256	+
IrGS17	2211354	2211194	2211637	443	2211348	2211365	-
IrGS18	2408285	2407962	2408633	671	2408258	2408275	+
IrGS19	2536664	2536348	2536952	604	2536659	2536676	-
IrGS20	2598060	2597782	2598307	525	2598065	2598082	-
IrGS21	2599974	2599592	2600285	693	2599980	2599997	-
IrGS22	2663968	2663791	2664228	437	2663970	2663987	-
IrGS23	2760688	2760531	2760982	451	2760719	2760736	-
IrGS24	2815385	2815201	2815649	448	2815411	2815428	-
IrGS25	2870433	2870115	2870780	665	2870430	2870447	-
IrGS26	2978162	2977878	2978494	616	2978165	2978182	-
IrGS27	3075166	3075122	3075406	284	3075159	3075176	+
IrGS28	3195552	3195195	3195949	754	3195564	3195581	+
IrGS29	3266581	3266380	3266891	511	3266593	3266610	-
IrGS30	3276693	3266378	3266885	507	3276695	3276712	+
IrGS31	3386998	3386860	3387132	272	3387010	3387027	-
IrGS32	3448213	3448017	3448476	459	3448221	3448238	-
IrGS33	3491164	3490926	3491476	550	3491151	3491168	-
IrGS34	3558532	3558221	3558693	472	3558533	3558550	+
IrGS35	3621908	3621546	3622192	646	3621918	3621935	-
IrGS36	3658670	3658562	3658860	298	3658708	3658725	+
IrGS37	3674947	3674567	3675021	454	3674951	3674968	-
IrGS38	3948038	3947754	3948265	511	3948134	3948151	-
IrGS39	4007370	4007008	4007710	702	4007345	4007362	-
IrGS40	4106580	4106396	4106740	344	4106583	4106600	-
IrGS41	4186557	4186458	4186734	276	4186583	4186600	-
IrGS42	4216234	4215902	4216613	711	4216231	4216248	+
IrGS43	4341322	4341079	4341614	535	4341310	4341327	+
IrGS44	4441905	4441537	4442335	798	4441909	4441926	-
IrGS45	4448335	4447488	4448133	645	4448337	4448354	-
IrGS46	4561060	4560829	4561192	363	4561068	4561085	-
IrGS47	4584577	4584383	4584779	396	4584622	4584640	+
IrGS48	4602274	4602126	4602555	429	4602283	4602300	+
IrGS49	4612122	4611914	4612371	457	4612121	4612138	-

Intragenic Antisense (D sites)

Peak	PC	PS	PE	PL	MS	ME	Strand(+/-)
IrGAs1	128696	128547	128941	394	128693	128710	-
IrGAs2	318299	318042	318459	417	318307	318325	-
IrGAs3	549836	549434	550118	684	549839	549856	+
IrGAs4	630656	630485	630727	242	774332	774349	-
IrGAs5	1053922	1053559	1054157	598	1053905	1053922	+
IrGAs6	1099750	1099614	1099867	253	1099724	1099741	+
IrGAs7	1204307	1204216	1204485	269	1204262	1204451	-
IrGAs8	1265403	1265110	1265727	617	1265417	1265434	-
IrGAs9	1357208	1357023	1357495	472	1357220	1357237	-
IrGAs10	1894351	1894135	1894739	604	1894347	1894364	-
IrGAs11	2058280	2057954	2058596	642	2058241	2058258	-
IrGAs12	2760688	2760450	2760865	415	2760967	2760985	+
IrGAs13	3195552	3195195	3195949	754	3195565	3195582	-
IrGAs14	3296918	3296428	3297079	651	3296920	3296937	-
IrGAs15	3347078	3346748	3347300	552	3347061	3347078	+
IrGAs16	3383005	3382401	3383131	730	3383011	3383028	-
IrGAs17	3435112	3434807	3435413	606	3435121	3435138	-
IrGAs18	3603190	3602998	3603508	510	3603188	3603205	-
IrGAs19	3621908	3621546	3622192	646	3621895	3621912	+
IrGAs20	3984487	3984181	3984915	734	3984481	3984498	+
IrGAs21	4135862	4135537	4136232	695	4135846	4135863	+
IrGAs22	4186557	4186122	4186896	774	4186572	4186589	+
IrGAs23	4288435	4287803	4288174	371	4288431	4288448	-
IrGAs24	4309656	4309597	4309829	232	4309647	4309664	+
IrGAs25	4371420	4371239	4371617	378	4371412	4371429	+

IGS=Intergenic Sense
IGAs=Intergenic Antisense
IrGS=Intragenic Sense
IrGAs=Intragenic Antisense
PC= Peak Center base pair
PS=Peak Start
PE=Peak End
PL= Peak Length
MS=Motif Start
ME= Motif End
