# Supplementary material for: Genome-Scale Mapping Reveals Complex Regulatory Activities of RpoN in Yersinia pseudotuberculosis
Source: mSystems. 2020 Nov 10;5(6):e01006-20. doi: 10.1128/mSystems.01006-20 (PMC7657599; doi:10.1128/mSystems.01006-20)
Supplement: TABLE S2 [file mSystems.01006-20-st002.pdf]

Table S2: Low confidence peaks of Rpon binding site

Peak		PC	PSL	PSI	PSO	CDS	Downstream gene	PSSM	Motif(+/-)	Motif Sequence
LCP1	C	41062	2.08	NA	NA	YPK_0038	YPK_0039	5.75	+	CCGGTACGGATATTGCGT
LCP2	C	88103	2.57	NA	NA	YPK_0077	YPK_0078	4.36	-	TTGGCAGTAATTTTGCTG
LCP3	C	209084	2.34	NA	NA	YPK_0161	YPK_0160	3.26	-	TGGGTCCGTTTCTTGCTC
LCP4	D	312934	1.77	1.77	NA	YPK_0275	YPK_0276	2.55	+	ATGGCAACGATTAACCAG
LCP5	A	319160	NA	2.02	NA	NA	YPK_0282,YPK_283	6.08	+	CTGGCAAATTCTTGCGTAG
LCP6	A	383821	2.30	4.31	NA	NA	YPK_0358	2.99	+	ACTCCCTATAATGCGCAT
LCP7	C	465181	2.58	NA	NA	YPK_0426	YPK_0425	3.90	-	TAAGCTTAAAGAATGCAT
LCP8	A	485490	3.34	5.35	3.88	NA	YPK_0444	3.70	-	TTATCATAGATATCCCTT
LCP9	B	573233	2.46	3.14	NA	NA	YPK_0523	4.44	-	CTGGCACTATCAAAGTTT
LCP10	C	694997	NA	3.50	NA	YPK_0636	YPK_0637	2.76	+	TCGAAATGAAAAAACCAC
LCP11	C	774341	3.39	NA	NA	YPK_0712	YPK_0713, YPK_0714	6.40	+	TTGGTGAAGAAGCGGCC
LCP12	C	860742	2.10	NA	NA	YPK_0763	YPK_0764	4.64	+	GCGGCACAGATATTGCCG
LCP13	C	3857936	NA	3.62	NA	YPK_3512	NA	5.12	+	TTGGCAGGGTTAGGGATT
LCP14	C	4195472	NA	7.02	NA	YPK_3807	NA	4.74	+	TCGGTACGGGGTGCGCAT
LCP15	D	4592326	1.75	NA	NA	YPK_4161	YPK_4160	4.36	-	TTGGATCTGTTCGCGCAT
LCP16	C	4659045	1.67	NA	NA	YPK_4219	NA	4.83	-	GCGGCAAGCAAACAGCAT

LCP = Low Confidence Peak, peak strength <2.5 and motif score <7.

Type= Type of binding site according to the position and direction of motif

PC= Peak Center base pair

PSL=Peak Strength Logarithmic phase

PSI=Peak Strength Virulence Inducing condition

PSO=Peak Strength Over-expression of Δ*rpoN*

PSSM= Position Specific Score Matrix
