# Supplementary material for: Genome-Scale Mapping Reveals Complex Regulatory Activities of RpoN in Yersinia pseudotuberculosis
Source: mSystems. 2020 Nov 10;5(6):e01006-20. doi: 10.1128/mSystems.01006-20 (PMC7657599; doi:10.1128/mSystems.01006-20)
Supplement: TABLE S3 [file mSystems.01006-20-st003.pdf]

TableS3: Global regulators differentially expressed in Δ*rpoN*

Locus tag	Gene Name	Description	Stationary phase		Induction condition	
			log2FC	*P*-adj value	log2FC	*P*-adj value
YPK_0452	*fis*	Transcriptional regulator, Fis	0.5	0.0146	4.3	0.0000
YPK_0634	*rpoD*	RNA polymerase sigma factor RpoD (σ^{70})	-0.1	0.1663	2.3	0.0000
YPK_1182	*rpoE*	RNA polymerase sigma factor RpoE (σ^{24})	-0.5	0.0000	-2.1	0.0000
YPK_2269	*luxR*	Two component LuxR family, FimZ	-0.1	0.5094	-1.8	0.0000
YPK_3425	*rpoS*	RNA polymerase sigma factor RpoS (σ^{38})	0.4	0.0000	-1.3	0.0000
YPK_3976	*rpoH*	RNA polymerase factor RpoH (σ^{32})	0.6	0.0000	2.6	0.0000
YPK_4132	*cpxR*	DNA-binding transcriptional regulator, CpxR	0.9	0.0000	2.0	0.0000
YPK_0001	*dnaA*	Chromosomal replication initiation protein, DnaA	0.7	0.0000	-0.1	0.8541
YPK_0248	*crp/fnr*	CRP/FNR family transcriptional regulator, Crp	-0.59	0.0000	-1.61	0.0000
YPK_1745	*flhD*	Transcriptional activator FlhD	-2.2	0.0000	-1.8	0.0000
YPK_1746	*flhC*	Transcriptional activator FlhC	-1.9	0.0000	-1.4	0.0000
YPK_1876	*rovA*	Transcriptional regulator, SlyA	-2.9	0.0000	-1.9	0.0000
YPK_1902	*tyrR*	DNA-binding transcriptional regulator, TyrR	0.0	0.8662	0.8	0.0010
YPK_2074	*hns*	DNA-binding protein, H-NS	-0.08	0.6141	-0.85	0.0078
YPK_2380	*fliA*	RNA polymerase sigma factor RpoD (σ^{28})	-2.97	0.0000	-1.74	0.0001
YPK_2385	*araC*	AraC family transcriptional regulator, AraC	-1.7	0.0000	-1.4	0.0001
YPK_3223	*asnC*	Lrp/AsnC family transcriptional regulator, AsnC	-0.63	0.0018	-0.57	0.0135
YPK_3372	*csrA*	Carbon storage regulator, CsrA	-0.12	0.5625	0.13	0.6673
YPK_3606	*arcA*	Aerobic respiration control protein, ArcA	-0.28	0.0790	-0.27	0.8555
YPK_3731	*nusA*	Termination/antitermination protein, NusA	0.0	0.9407	1.4	0.0000
YPK_4034	*rho*	Transcription termination factor, Rho	0.5	0.0000	1.4	0.0000
