# Supplementary material for: Genome-Scale Mapping Reveals Complex Regulatory Activities of RpoN in Yersinia pseudotuberculosis
Source: mSystems. 2020 Nov 10;5(6):e01006-20. doi: 10.1128/mSystems.01006-20 (PMC7657599; doi:10.1128/mSystems.01006-20)
Supplement: TABLE S4 [file mSystems.01006-20-st004.pdf]

Table S4: Validations of productive RpoN binding

Group#	Locus tag*	Gene function	Expression compared to *Y. pseudotuberculosis* WT	
			Δ*rpoN*	Binding site mutant
A	YPK_0329	N-acetyltransferase	↙	↙
	YPK_1886	Putative tripeptide transporter permease, TppB	↗	↗
	YPK_1894	Phage shock protein, PspA	↙	↙
	YPK_2909	Zinc resistance protein, ZraP	↗	↗
	YPK_2908	Sensor kinase, ZraS	↙	↙
	YPK_2927	Asparagine synthetase B, AsnB	↙	–
	YPK_3010	Glutamate and aspartate transporter subunit, GltI	↙	↙
D	YPK_2997	Glucosamine-6-phosphate deaminase, NagB	↗	↗
	YPK_3962	Glycerol-3-phosphate transporter , UgpB	↗	↗
	YPK_3075	2,5-didehydrogluconate reductase, DkgA	↗	↗
	YPK_3613	Soluble lytic murein transglycosylase, Slt	↗	↗
	YPK_3887	NmrA family protein	↗	–

grouping of binding sites depending on position (A) intergenic sense strand (D) intragenic anti sense strand

*locus tag for downstream gene (A) or gene on corresponding sense strand

↙ = Down-regulated in Δ*rpoN*/WT

↗ = Up-regulated in RpoN binding site mutant/WT

– = No change in relative expression
