# Supplementary material for: Genome-Scale Mapping Reveals Complex Regulatory Activities of RpoN in Yersinia pseudotuberculosis
Source: mSystems. 2020 Nov 10;5(6):e01006-20. doi: 10.1128/mSystems.01006-20 (PMC7657599; doi:10.1128/mSystems.01006-20)
Supplement: TABLE S5 [file mSystems.01006-20-st005.pdf]

Table S5: List of strains used in this study

Strain or plasmid	Relevant phenotype	Reference	RpoN binding site mutation
	E. coli		
S17-1 λ-pir	*recA, thi, pro, hsdRM+* ,SmR , <RP4:2-Tc:Mu:Km:Tn7>TpR		
pUC57-3xV5 / DH5α	pUC 57 derivative containing a 129bp fragment of 3xV5	This study	
	Y. pseudotuberculosis		
YpIII/pIBX (wt)	YpIII strain with Tn5*luxCDABE* inserted in the Tn1000 resolvase homolog in pCD1 (wild type); KmR	Caliper Life Sciences,	
YPIII,Δ*rpoN* /pIBX	In frame deletion of *rpoN* (YPK_0505); Km^r	This study	
YPIII,*rpoN* :V5/pIBX	*rpoN* tagged with 3xV5	This study	
YPIII,Δ*rpoN* /pIBX, p*rpoN*	Δ*rpoN* transcomplemented	This study	
YPIII,Δ*rpoN* /pIBX, p*rpoN* :V5	V5-overexpressing YPIII,ΔrpoN/pIBX strain	This study	
YPIII,YPK_0329_BS-mut/pIBX	Mutations in rpoN Binding Site upstream of YPK_0329	This study	t**gta**cttgtttat**gag**tt
YPIII,YPK_0464_BS-mut/pIBX	Mutations in rpoN Binding Site in YPK_0464	This study	at**ca**tccgtttatt**aa**g**a**
YPIII,YPK_1170_BS-mut/pIBX	Mutations in rpoN Binding Site in YPK_1170	This study	ca**a**gc**t**tggattat**t**caa
YPIII,YPK_1886_BS-mut/pIBX	Mutations in rpoN Binding Site upstream of YPK_1886	This study	tg**ta**cacgcattt**gag**ct
YPIII,YPK_1894_BS-mut/pIBX	Mutations in rpoN Binding Site upstream of YPK_1894	This study	t**cat**cacgactct**act**tt
YPIII,YPK_2431_BS-mut/pIBX	Mutations in rpoN Binding Site in YPK_2431	This study	tt**a**gc**t**cgcttc**c**t**aa**gc
YPIII,YPK_2908_BS-mut/pIBX	Mutations in rpoN Binding Site upstream of YPK_2908	This study	a**gta**catgataat**gag**tt
YPIII,YPK_2909_BS-mut/pIBX	Mutations in rpoN Binding Site upstream of YPK_2909	This study	t**gta**cataggaat**gag**ct
YPIII,YPK_2927_BS-mut/pIBX	Mutations in rpoN Binding Site upstream of YPK_2927	This study	t**gta**tccaagagt**gag**tt
YPIII,YPK_2997_BS-mut/pIBX	Mutations in rpoN Binding Site in YPK_2997	This study	t**gcca**ac**a**agatt**a**gcat
YPIII,YPK_3010_BS-mut/pIBX	Mutations in rpoN Binding Site upstream of YPK_3010	This study	c**gta**cacaaacct**gag**at
YPIII,YPK_3075_BS-mut/pIBX	Mutations in rpoN Binding Site in YPK_3075	This study	at**a**gc**t**c**t**gat**c**tt**a**ccc
YPIII,YPK_3583_BS-mut/pIBX	Mutations in rpoN Binding Site in YPK_3583	This study	c**a**gg**a**cc**t**catga**c**g**ga**g
YPIII,YPK_3613_BS-mut/pIBX	Mutations in rpoN Binding Site in YPK_3613	This study	ctg**tga**taaatc**c**tg**t**gt
YPIII,YPK_3887_BS-mut/pIBX	Mutations in rpoN Binding Site in YPK_3887	This study	c**gcat**tcaat**c**aa**a**gcat
YPIII,YPK_3962_BS-mut/pIBX	Mutations in rpoN Binding Site in YPK_3962	This study	g**acca**gcatt**g**tt**a**gcat
	**Plasmids**		
pDM4	Suicide vector for construction of deletion and Binding site mutants; *sacBR; oriR6K* ; CmR	Milton et al., 1996	
p*rpoN*	*rpoN* under araBAD promoter in pBAD24; CbR	This study	
p*rpoN:V5*	*rpoN* C-terminal tagged with 3xV5 under the araBAD promoter in pBAD18; CbR	This study	
pDM4-YPK_0505	The flanking DNA regions of the YPK_0505 gene were PCR amplified from *pseudotuberculosis* genome using primers xxxx and cloned in the pDM4 *Y.* plasmid by SacI-XhoI restriction	This study	
pDM4-0329_BS-mut	The mutated rpoN Binding Site in YPK_0329 including flanking regions were cloned in the pDM4 plasmid by SacI-XhoI restriction	This study	
pDM4-0464_BS-mut	The mutated rpoN Binding Site in YPK_0464 including flanking regions were cloned in the pDM4 plasmid by SacI-XhoI restriction	This study	
pDM4-1170_BS-mut	The mutated rpoN Binding Site upstream of YPK_1170 including flanking regions were cloned in the pDM4 plasmid by SacI-XhoI restriction	This study	
pDM4-1886_BS-mut	The mutated rpoN Binding Site in YPK_1886 including flanking regions were cloned in the pDM4 plasmid by SacI-XhoI restriction	This study	
pDM4-1894_BS-mut	The mutated rpoN Binding Site upstream of YPK_1894 including flanking regions were cloned in the pDM4 plasmid by SacI-XhoI restriction	This study	
pDM4-2431_BS-mut	The mutated rpoN Binding Site in YPK_2431 including flanking regions were cloned in the pDM4 plasmid by SacI-XhoI restriction	This study	
pDM4-2908_BS-mut	The mutated rpoN Binding Site upstream of YPK_2908 including flanking regions were cloned in the pDM4 plasmid by SacI-XhoI restriction	This study	
pDM4-2909_BS-mut	The mutated rpoN Binding Site upstream of YPK_2909 including flanking regions were cloned in the pDM4 plasmid by SacI-XhoI restriction	This study	
pDM4-2927_BS-mut	The mutated rpoN Binding Site in YPK_2927 including flanking regions were cloned in the pDM4 plasmid by SacI-XhoI restriction	This study	
pDM4-2997_BS-mut	The mutated rpoN Binding Site in YPK_2997 including flanking regions were cloned in the pDM4 plasmid by SacI-XhoI restriction	This study	

pDM4-3010_BS-mut	The mutated rpoN Binding Site upstream of YPK_3010 including flanking regions were cloned in the pDM4 plasmid by SacI-XhoI restriction	This study
pDM4-3075_BS-mut	The mutated rpoN Binding Site upstream of YPK_3075 including flanking regions were cloned in the pDM4 plasmid by SacI-XhoI restriction	This study
pDM4-3583_BS-mut	The mutated rpoN Binding Site in YPK_3583 including flanking regions were cloned in the pDM4 plasmid by SacI-XhoI restriction	This study
pDM4-3613_BS-mut	The mutated rpoN Binding Site in YPK_3613 including flanking regions were cloned in the pDM4 plasmid by SacI-XhoI restriction	This study
pDM4-3887_BS-mut	The mutated rpoN Binding Site in YPK_3887 including flanking regions were cloned in the pDM4 plasmid by SacI-XhoI restriction	This study
pDM4-3962_BS-mut	The mutated rpoN Binding Site in YPK_3962 including flanking regions were cloned in the pDM4 plasmid by SacI-XhoI restriction	This study
