# Supplementary material for: Genome-Scale Mapping Reveals Complex Regulatory Activities of RpoN in Yersinia pseudotuberculosis
Source: mSystems. 2020 Nov 10;5(6):e01006-20. doi: 10.1128/mSystems.01006-20 (PMC7657599; doi:10.1128/mSystems.01006-20)
Supplement: TABLE S6 [file mSystems.01006-20-st006.pdf]

Table S6: Primers used in this study

Primer name	Sequence (5' to 3')	Vector/Template
A.del.rpoN.Xho	ACCGTCGACCCTCGAGGGTTGACCCAATTTCAGTGATC	pDM4/YPIII
B.del.rpoN	GTTCAGGTCAAACCAGACCTTGCTTCATAATGCTGTATC	pDM4/YPIII
C.del.rpoN	TTATGAAGCAAGGTCTGGTTTGACCTGAACTGAGAA	pDM4/YPIII
D.del.rpoN.Sac	TGGAATTCCCGGGAGAGCTCGAGTGTAGAACCTTTGCTGAGTA	pDM4/YPIII
Kpn.rpoN.F	GCGAATTCGAGCTCGGTACCAGGAGGAAACGATGAAGCAAGGTCTGCAAC	pBAD18/YPIII
Sal.rpoN.R	TAGGCTTACCGTCGACAACCAACTGTTTACGTTGAT	pBAD18/YPIII
Sal.V5.F	GTAAACAGTTGGTTGTCGACGGTAAGCCTATCC	pBAD18/pUC57:3xV5
Xho.V5.R	ATGCCTGCAGGTCGAGTCACGTGCTGTCAA	pBAD18/pUC57:3xV5
A.rpoN.V5.Sac	TGGAATTCCCGGGAGAGCTCAGGCTGTGGATATGCATG	pDM4/pBAD18.rpoN:3xV5
B.rpoN.V5	TCTCAGTTCAGGTCACGTGCTGTCAAGGCCAAG	pDM4/pBAD18.rpoN:3xV5
C.rpoN.V5	GGCCTTGACAGCACGTGACCTGAACTGAGAAGGAATAG	pDM4/YPIII
D.rpoN.V5.Xho	ACCGTCGACCCTCGACCTTTGCTGAGTAATAACAGC	pDM4/YPIII
0329_BS.A	ACCGTCGACCCTCGAGTCTACCAACTGAGCTATGCC	pDM4/YPIII
0329_BS.B	CTCATAAACAAGTACAACTTATAAAGTGGGCAGTAGTAA	pDM4/YPIII
0329_BS.C	GTACTTGTTTATGAGTTAGCAATGGATATCAGAGCAGAC	pDM4/YPIII
0329_BS.D	TGGAATTCCCGGGAGAGCTCTTATGTCCTATCCCAGTGCG	pDM4/YPIII
0464_BS.A	ACCGTCGACCCTCGAGCCCGTTATTACTGTTGATGGC	pDM4/YPIII
0464_BS.B	TCTTAATAAACGGATGATTTGGCGGATTTCTTCGAT	pDM4/YPIII
0464_BS.C	CATCCGTTTATTAAGAGGCGTAGGCTGCAAAGTG	pDM4/YPIII
0464_BS.D	TGGAATTCCCGGGAGAGCTCTCAACGATCAGGCTAACATG	pDM4/YPIII
1170_BS.A	ACCGTCGACCCTCGAGTTCAGCAGGAAGGCATCA	pDM4/YPIII
1170_BS.B	TGAATAATCCAAGCTTGCGCCCGCTCTTTCAAA	pDM4/YPIII
1170_BS.C	AGCTTGGATTATTCAAATTAAAAGTGCGACATCAGGC	pDM4/YPIII
1170_BS.D	TGGAATTCCCGGGAGAGCTCAGATAATCGCCCCGAAAG	pDM4/YPIII
1886_BS.A	ACCGTCGACCCTCGAGCGCTTTAATCATCAGCATGT	pDM4/YPIII
1886_BS.B	GCTCAAATGCGTGTACACAAAAATAGTTGTGTAAATTTTT	pDM4/YPIII
1886_BS.C	TACACGCATTTGAGCTATTCTGCCTCGCCCCTG	pDM4/YPIII
1886_BS.D	TGGAATTCCCGGGAGAGCTCAGATGATTAGCGGCGGTT	pDM4/YPIII
1894_BS.A	ACCGTCGACCCTCGAGGCTTCATGGCCGAATAAC	pDM4/YPIII
1894_BS.B	CTCAGAGTCGTGTACACTTTTCATGATAATTAATAGCATG	pDM4/YPIII
1894_BS.C	GTACACGACTCTGAGTTAACTCTATGTGAAGCGTATTG	pDM4/YPIII
1894_BS.D	TGGAATTCCCGGGAGAGCTCGTTTTAGCGTCTCAACTAAC	pDM4/YPIII
2431_BS.A	ACCGTCGACCCTCGAGGCATAACTTCACTTGCAAACC	pDM4/YPIII
2431_BS.B	TAGCTCGCTTCCTAAGCCGTAGCTTGCCGCAAAT	pDM4/YPIII
2431_BS.C	TTAGGAAGCGAGCTAATAATGCCCGTAGTGCATGATTAAC	pDM4/YPIII
2431_BS.D	TGGAATTCCCGGGAGAGCTCCGCACCATTATAGAAACACTGG	pDM4/YPIII
2908_BS.A	ACCGTCGACCCTCGAGGGTCCTTAATCGCCATAG	pDM4/YPIII
2908_BS.B	GTACATGATAATGAGTTTAAATAATGGTTGTTTTTTTAG	pDM4/YPIII
2808_BS.C	CTCATTATCATGTACTTAGCATATTGGCATAGGAAT	pDM4/YPIII
2808_BS.D	TGGAATTCCCGGGAGAGCTCCATGGAATTCTTTCCAGAGT	pDM4/YPIII
2809_BS.B	CTCATTCCTATGTACATATGCTAATGGCATGATAATTGC	pDM4/YPIII
2809_BS.C	GTACATAGGAATGAGCTTAACGTAATAAGCTTACTACG	pDM4/YPIII
2927_BS.A	ACCGTCGACCCTCGAGACTCAATCAACCCCACAGAG	pDM4/YPIII
2927_BS.B	CTCACTCTTGGATACAGTTGCCGAACACAGAAGTA	pDM4/YPIII
2927_BS.C	GTATCCAAGAGTGAGTTGATCTCTGTAGATTATCTGAAGG	pDM4/YPIII
2927_BS.D	TGGAATTCCCGGGAGAGCTCGCTCCAGATTGATATCCCAG	pDM4/YPIII
2997_BS.A	ACCGTCGACCCTCGAGTACGTTGGCTTGCCGAAA	pDM4/YPIII
2997_BS.B	TTGCCAACAAGATTAGCATCACCACCAAAGAAACG	pDM4/YPIII
2997_BS.C	TAATCTTGTTGGCAAATATGCATTGACGGTTGGG	pDM4/YPIII
2997_BS.D	TGGAATTCCCGGGAGAGCTCATCAGCAACGACAACAGC	pDM4/YPIII
3010_BS.A	ACCGTCGACCCTCGAGGGTTTGGTCACTCCATTG	pDM4/YPIII
3010_BS.B	CTCAGGTTTGTGTACGTAGGGGGAGAACTAAGC	pDM4/YPIII
3010_BS.C	GTACACAAACCTGAGATTATTTCTACTCATAGTATG	pDM4/YPIII
3010_BS.D	TGGAATTCCCGGGAGAGCTCTCTTTCCTAATGGCATCCAC	pDM4/YPIII
3583_BS.A	ACCGTCGACCCTCGAGCCAGTGGCGCTAAAACAGTA	pDM4/YPIII
3583_BS.B	TCCGTCATGAGGTCCTGGGCTGGCTTCTGGATGC	pDM4/YPIII
3583_BS.C	GGACCTCATGACGGAGCGCCATTGTTTGATCATTTTATC	pDM4/YPIII

3583_BS.D	TGGAATTCCCGGGAGAGCTCAAGTTGCATCGGCCATTTCC	pDM4/YPIII
3887_BS.A	ACCGTCGACCCTCGAGTCGGCTGATCATTGACAC	pDM4/YPIII
3887_BS.B	CGCATTCAATCAAAGCATAAGCTTGATAATATGTCGCG	pDM4/YPIII
3887_BS.C	CTTTGATTGAATGCGGATTAGCCCGTAAGGTAAAAATG	pDM4/YPIII
3887_BS.D	TGGAATTCCCGGGAGAGCTCCGTCATTCATACCCTTGG	pDM4/YPIII
3962_BS.A	ACCGTCGACCCTCGAGCTCTTCATCAACCACGGT	pDM4/YPIII
3962_BS.B	GCTAACAATGCTGGTCAAAACGCCATTATTGGTGG	pDM4/YPIII
3962_BS.C	ACCAGCATTGTTAGCATCTGCGTCATACGGCAT	pDM4/YPIII
3962_BS.D	TGGAATTCCCGGGAGAGCTCGGGTTCTAGTTGCGGTTA	pDM4/YPIII
3613_BS.A	ACCGTCGACCCTCGAGCAACTGCCCGGTAATGACT	pDM4/YPIII
3613_BS.B	CACAGGATTTATCACAGGTGAGTACGGCGCAAG	pDM4/YPIII
3613_BS.C	GTGATAAATCCTGTGTCAACTGACGGTATTCCAAATAAG	pDM4/YPIII
3613_BS.D	TGGAATTCCCGGGAGAGCTCTCTTACTGCCTATCTGCGAC	pDM4/YPIII
3075_BS.A	ACCGTCGACCCTCGAGGAAACGCAAGTAGGGAATGC	pDM4/YPIII
3075_BS.B	TAGCTCTGATCTTACCCGCCTGGTGTAATTCTTC	pDM4/YPIII
3075_BS.C	GTAAGATCAGAGCTATTGGCGTCAGTAACTTTCATC	pDM4/YPIII
3075_BS.D	TGGAATTCCCGGGAGAGCTCCCAGCGTAATACAACCTGTC	pDM4/YPIII
